# Integrating spectral signatures and microbial profiling to differentiate diseased and healthy corals in the Red sea

**DOI:** 10.1038/s41598-026-50675-z

**Published:** 2026-05-18

**Authors:** Ahmed M. Khalifa, Khaled Z. ElBaghdady, Moaz M. Hamed, Ashraf S. Mohammad, Mostafa A. Khaled

**Affiliations:** 1 Marine Sciences Department, National Authority for Remote Sensing and Space Sciences (NARSS), Cairo, Egypt; 2 Microbiology Department, Faculty of Science, Ain Shams University, Cairo, Egypt; 3 National Institute of Oceanography and Fisheries (NIOF), Hurghada, Egypt

**Keywords:** Coral reef diseases, Bacterial spectral signatures, Coral-associated microbiome, Red sea, Hyperspectral analysis, Coral disease detection, Biological techniques, Ecology, Ecology, Microbiology

## Abstract

Coral reef ecosystems face increasing threats from microbial diseases, especially those induced by bacterial infections. Conventional diagnostic techniques often require invasive sampling, extended processing time and are limited in their spatial applicability. Spectral reflectance analysis offers a non-invasive means for detecting subtle physiological alterations associated with coral disease; however, its application in characterizing microbiological changes remains largely unexplored. This research aimed to differentiate healthy from diseased coral colonies by analyzing the spectral fingerprints of the disease and their associated bacterial communities, using hyperspectral data, microbial profiling, and multivariate statistical analysis. The bacterial species identified in healthy coral samples included *Bacillus subtilis*, *Cytobacillus firmus*, *Bacillus amyloliquefaciens*, and *Bacillus sporothermodurans*. In contrast, the bacteria associated with diseased coral samples were *Vibrio pelagius* and *Vibrio fortis*. Healthy corals demonstrate consistently lower reflectance across all bands in comparison to diseased corals. The reflectance of diseased *Favia lacuna* showed a notable increase when compared to healthy specimens, especially at wavelengths of 594 nm, 649 nm, and 702 nm. In contrast, *Acropora humilis* exhibited heightened peaks at wavelengths of 580 nm, 693 nm, and 702 nm. The analysis of the second derivative revealed that coral colonies affected by disease exhibited distinct negative peaks at wavelengths of 450–460 nm, 580–590 nm, and 700–800 nm. The identified peaks are likely associated with tissue thinning, skeletal exposure, or microbial biofilm accumulation rather than pigment absorption, given that this region is dominated by scattering effects. In contrast, healthy colonies exhibited stable characteristics at approximately 675 nm, indicating the presence of intact symbiotic chlorophyll and preserved physiological structure. The present study demonstrates that hyperspectral reflectance profiling of bacterially infected corals shows promising potential as a non-invasive approach for differentiating healthy and diseased coral microbiomes. The integration of spectral indicators with microbial community data provides preliminary insights into coral health assessment and may contribute to the development of improved strategies for disease detection and understanding coral–microbe interactions under environmental stress.

**Supplementary Information:**

The online version contains supplementary material available at 10.1038/s41598-026-50675-z.

## Introduction

 Coral reefs, including over a million marine species, provide essential ecosystem services such as fisheries, tourism, and shoreline protection, making them highly varied and ecologically valuable ecosystems^1^. Approximately 25% of marine life depends on coral reefs for habitat, highlighting their importance for marine biodiversity^2^. Coral reef diseases pose a significant danger to the sustainability and ecological integrity of these essential systems. Diseases serve a major role in coral population decline, and climate change makes coral health problems worse, which affects biodiversity and ecological services. Implementing successful conservation strategies requires knowledge of various diseases and their effects on the environment. Significant diseases affecting coral populations include aspergillosis, stony coral tissue loss disease, black band disease, and white band disease. Coral diseases exacerbate the decline of crucial keystone species, thereby diminishing biodiversity and altering community structures^3^.

Coral health is seriously threatened by climate change, which emphasizes the need for creative ways to identify stressors and diseases early on. Non-invasive monitoring techniques include photogrammetry and optical methods. While causing the least amount of physical disturbance, optical techniques like multispectral satellite surveys and hyperspectral imaging provide important information about coral health. By examining the physiology and structure of reefs, photogrammetry technology advances our knowledge of coral ecosystems^4,5^. Hyperspectral imaging has transformed remote sensing into a potent instrument for analyzing the spectral signatures associated with coral health conditions. This technology allows rapid, non-invasive assessments of coral health in response to the increasing incidence of coral bleaching and diseases. Hyperspectral imaging, which captures reflectance and fluorescence spectra, enables the identification of healthy corals and those under stress. Peaks linked to chlorophyll and fluorescent proteins act as indicators of health status^6,7^.

Research indicates that hyperspectral data can differentiate between asymptomatic and diseased corals, enhancing traditional visual diagnostic methods^8^. Coral diseases may severely impact reef ecosystems; however, by comprehending the microbial signature, we can develop novel therapies and facilitate early detection. The microbiology of diseases in corals is significantly influenced by the dynamics of bacterial communities, particularly the behavior of potential pathogens. Numerous bacterial species, particularly those within the *Vibrio* genus, significantly contribute to coral disease, despite the mitigating effects of quorum sensing (QS) on virulence factors. This relationship underscores the significance of comprehending microbial interactions within the context of coral damage and health status. *Vibrio coralliilyticus* utilizes quorum sensing to regulate approximately 300 virulence-associated genes, including those involved in biofilm formation and protease production, thereby facilitating effective colonization and infection^9^.

The Red Sea proposes a unique environment for the examination of coral reef ecosystems, characterized by significant environmental gradients and extreme conditions^10^. The Egyptian Red Sea, characterized by its established yet locally understudied microbiome diversity and notable coral resilience under extreme temperature and salinity conditions, serves as an ideal testing location^11^. Recent research conducted in the Red Sea has shown that coral mucus is home to dynamic bacterial communities that exhibit species-specific microbiome plasticity (for example, *Favia* spp. and *Pocillopora* spp.) across a variety of environmental gradients^11,12^. The optical characteristics of coral skeletons and tissues have been clarified via the use of hyperspectral imaging, which has shown strong connections between the pigment concentration and reflectance spectra^13^. Previous studies have employed hyperspectral methods to differentiate coral genera and benthic substrates in the Red Sea, indicating that pigment concentration and colony morphology significantly affect spectral signatures^14^. Research on Red Sea microbiomes indicates that bacterial communities exhibit host specificity and are organized according to environmental gradients, with prominent taxa such as Gammaproteobacteria and Vibrionaceae identified in a systematic review of 54 studies^10^. Nevertheless, limited studies have combined reflectance spectral analysis with microbial profiling for the purpose of disease detection in corals.

Significant knowledge gaps persist despite considerable advancements in our comprehension of coral microbiomes and the development of hyperspectral imaging techniques. Current research predominantly examines coral spectral characteristics or microbial diversity in isolation, without integrating both approaches for diagnostic applications. Research directly connecting bacterial composition to specific spectral biomarkers for coral disease detection is limited. Second derivative spectral analysis has been utilized in a limited number of studies to differentiate microbial infections in coral colonies, with even fewer successfully implementing this technique for such purposes. Since the late 1990 s, derivative spectroscopy, including second derivatives, has been employed to improve spectral separation. Holden and LeDrew’s study employed in-situ reflectance derivatives, both first and second order, to distinguish between healthy and bleached coral substrates. Nonetheless, these studies did not specifically address microbial pathogens or alterations in bacterial communities within corals^15,16^.

The use of second derivative analysis to differentiate bacterial infections in coral tissues is largely unexamined, highlighting the originality and importance of the present study. Furthermore, the ecological and biogeographical significance of the Egyptian Red Sea is not matched by the availability of microbial-level assessments of coral diseases in this area, which remain limited. This highlights the urgent requirement for interdisciplinary research that integrates microbial ecology and optical sensing to create non-invasive, rapid diagnostic tools for early disease detection in coral reef systems. Addressing these gaps is essential for facilitating rapid, field-deployable potential diagnostic capability of coral health. A non-invasive spectral-microbiome framework has the potential to transform coral disease monitoring by facilitating early detection of dysbiosis and pathogen outbreaks, thereby enhancing conservation strategies.

This study aims to investigate the integration of hyperspectral reflectance analysis of coral colonies with microbial community profiling of related bacteria to distinguish between healthy and unhealthy *Acropora* and *Favia* coral colonies in the Red Sea. To accomplish this, we delineate the mean and second-derivative spectral signatures of bacterial isolates, ascertain principal component loadings and spectral biomarkers, and utilize multivariate analyses such as PCA, Hierarchical Cluster Analysis, K-means clustering, Kruskal–Wallis tests, and Canonical Correspondence Analysis to investigate spectral-microbial patterns and their correlation with environmental variables. This method offers first proof-of-concept insights into the prospective use of spectral-microbial profiling as a non-invasive technique for evaluating coral health.

## Materials and methods

A concise methodological workflow is presented in a flowchart at the conclusion of this section (Fig. 3). The diagram offers a comprehensive outline of the study’s framework, commencing with field sampling and spectral measurements, followed by bacterial isolation and identification, spectral preprocessing, and subsequent multivariate analyses.

**Fig. 1:**
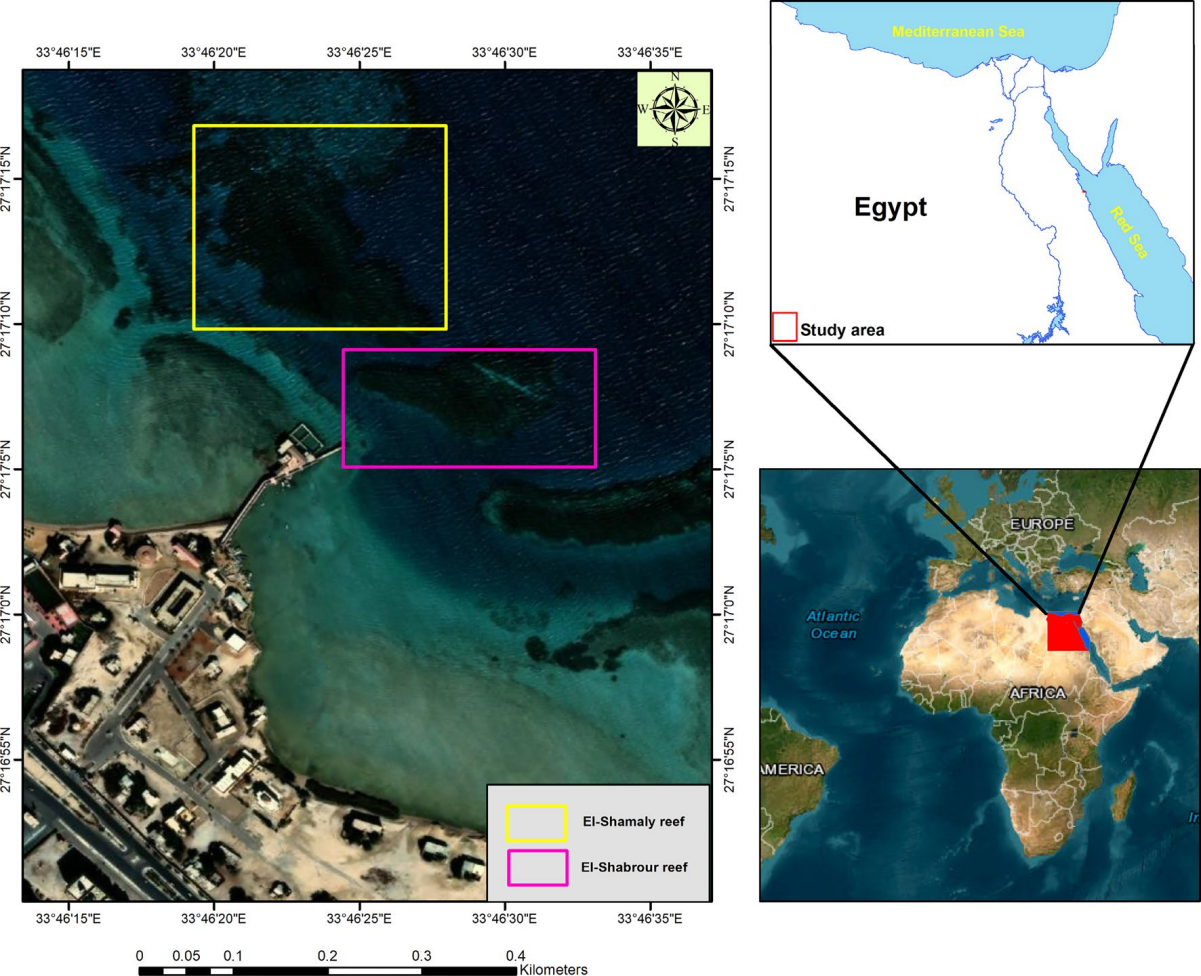
Georeferenced map of the study area along the Red Sea coast near Hurghada, Egypt, showing the locations of sampled coral reef sites surveyed for disease monitoring and spectral analysis. The map was generated using ArcGIS (version 10.7.1; https://www.esri.com).

### Study site and sample collection

To establish a systematic and consistent sampling methodology, the health state of the coral was first evaluated in the field before sample collection. Coral colonies were categorized as healthy or unhealthy according to a series of visual and physiological characteristics assessed in situ before sampling^17^. Healthy corals exhibited a well-preserved tissue structure, consistent coloration, and an absence of visible lesions or irregular patterns. In contrast, affected corals displayed signs such as tissue discoloration, tissue degradation, and/or necrotic lesions^18^. The established criteria ensured a consistent and reproducible classification of coral health status prior to the implementation of spectroscopic and microbiological investigations.

In February 2025, two reef patches, El-Shamaly and El-Shabrour, located on the Red Sea coast of Hurghada, Egypt (Fig. 1) (coordinates: 27° 17′ 7.28″ N, 33° 46′ 28.01″ E and 27° 17′ 13.74″ N, 33° 46′ 21.19″ E), were examined to assess the prevalence of diseased and healthy coral reefs. Eight samples were collected from both healthy and diseased portions of stony coral *Acropora* sp. and *Favia* sp. at a depth of approximately 1 to 4 m. At each sampling event, a coral fragment measuring approximately 2 cm was extracted from each coral colony using a chisel and hammer. The tissue layers of the corals were scraped from both healthy and diseased areas using a sterile dissecting knife. The coral fragments were transported in sterile filtered seawater within sterile 50 mL Falcon tubes, stored in an icebox, and promptly transferred to the microbiology laboratory at the National Institute of Oceanography and Fisheries (NIOF), Egypt. Conversely, in-situ water quality parameters such as temperature, pH, and salinity were assessed at each sampling location utilizing the Eureka Manta2 multi-probe water quality device (Eureka Environmental Engineering, USA).

**Fig. 2:**
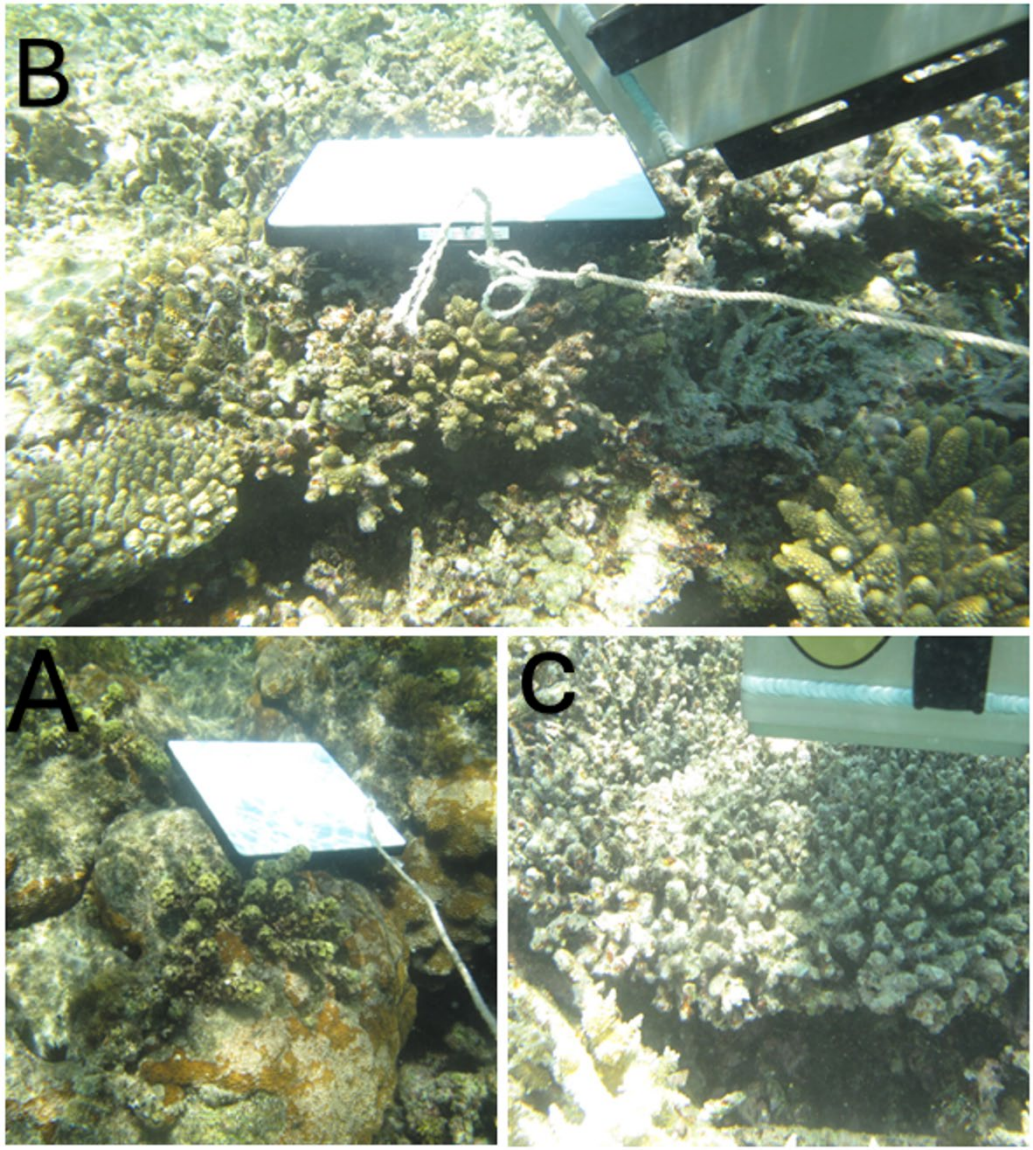
Field setup for in situ spectral measurements using the HR-512i spectroradiometer. **A** The calibrated white reference panel. **B** Acquisition of white reference reflectance over the sea-bottom substrate. **C** Radiance measurement from live coral colonies under natural light conditions.

### Spectral data collection

**Fig. 3:**
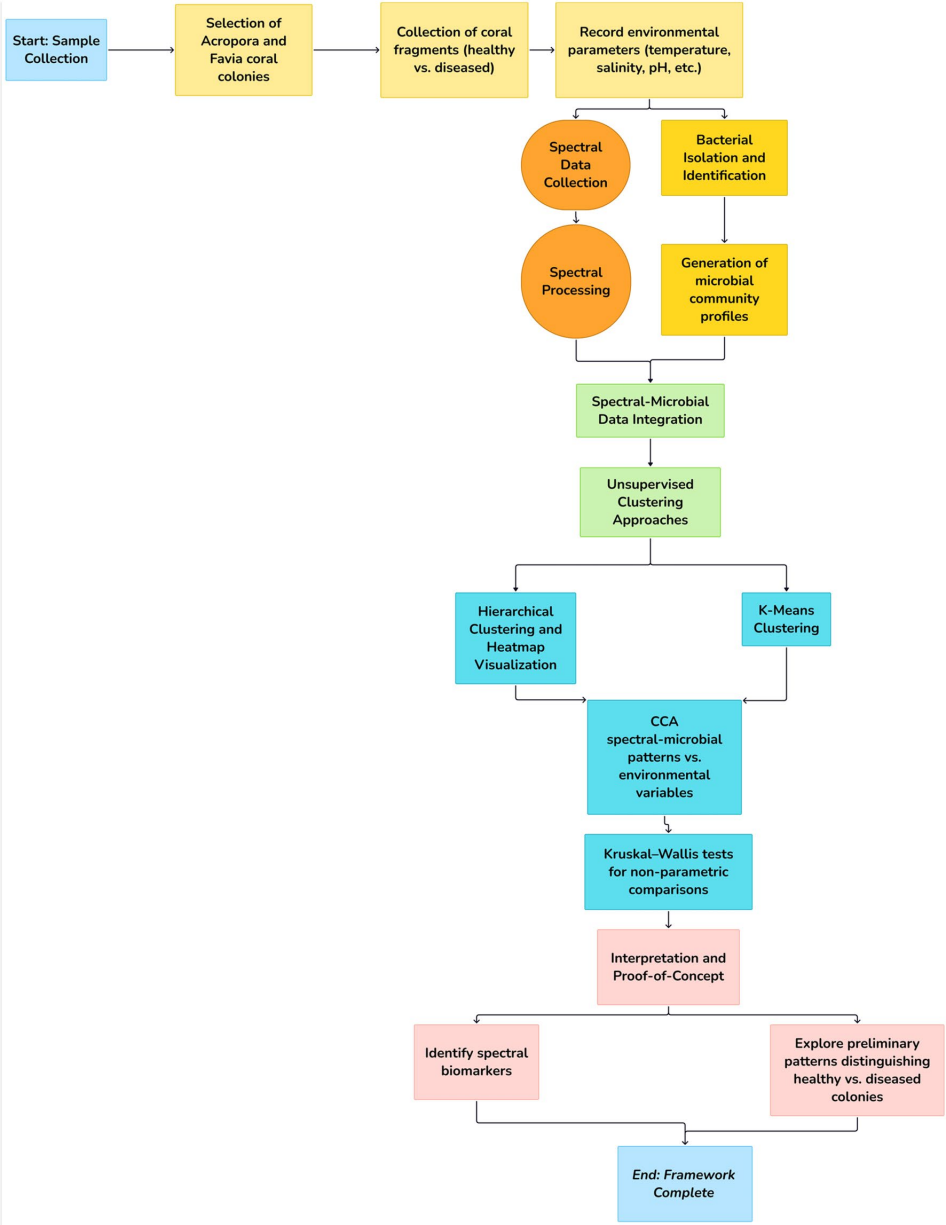
Flowchart of the methodological framework illustrates data collection, analysis, and interpretation steps.

The reflectance spectra of the specified coral reef species were obtained using the HR-512i underwater spectroradiometer (Fig. 2). This instrument combines two spectral channels to measure incident and reflected radiation fluxes from underwater benthic objects concurrently. Reflectance data were collected at the selected sites at 10:00 am and 12:00 pm during low tide and clear water conditions. This range facilitated comprehensive coverage of the coral surface while reducing water column effects, thereby ensuring precise data acquisition and consistency in the captured spectral signatures. The HR-512i spectroradiometer features a spectral range of 350 nm to 1050 nm and achieves a resolution of 3.2 nm at 700 nm, delivering high-precision spectral data. This device comprises a linear array of 512 channels and possesses a standard field of view (FOV) of 4 degrees. Reflectance measurements were performed directly using the spectroradiometer, thereby obviating the necessity for external cables. The instrument’s internal memory accommodates up to 1000 scans, enabling thorough data collection in the field. White reference measurements were conducted using a Spectralon white reference panel, composed of sintered polytetrafluoroethylene (PTFE), recognized for its near-Lambertian reflectance characteristics. Spectralon exhibits diffuse reflectance throughout the 350–2000 nm spectral range, achieving over 97% reflectance in most areas, thereby serving as an almost 100% reflective surface. The elevated uniform reflectance reduces spectral bias, thereby providing consistent calibration input for the spectroradiometer. In each measurement session, the spectroradiometer’s cosine receptor facilitated irradiance measurements, while a white reference panel exhibiting approximately 100% reflectance across the spectrum was utilized for instrument calibration (Fig. 3).

Spectral measurements of *Acropora* and *Favia* colonies were conducted exclusively at the tips of the coral colonies, as this region demonstrates the highest levels of bacterial activity and symptoms of disease. Concentrating on this region, it guarantees that the acquired spectral data are highly pertinent for distinguishing between diseased and healthy colonies, while reducing variability stemming from internal colony heterogeneity.

The spectroradiometer was calibrated before each measurement session using a Spectralon white reference panel to guarantee data precision. Calibration was conducted repeatedly to rectify any drift encountered during fieldwork. The gadget was situated around 10 cm from the coral surface, secured with a portable bracket to reduce movement, while all measurements were conducted immediately underwater. Environmental characteristics, including water depth, temperature, and ambient light conditions, were documented for each measurement to address possible variability.

To ensure data reliability, 5 to 10 spectral readings were collected for each coral species, thereby capturing intra-species variability. The collected spectra were monitored and stored directly on the device, facilitating immediate quality assessments and necessary adjustments. This structured sampling method ensures the robustness and reproducibility of the spectral data collected, while focusing on coral regions related to the detected bacterial disease. To reduce random variability, replicate spectra were averaged following the exclusion of outliers that exceeded ±2 standard deviations from the mean reflectance, utilizing the SVC HR-512i software. Spectral peaks were validated by cross comparing their positions and shapes across replicates to ensure signal consistency prior to derivative transformation and multivariate analysis.

### Spectral analysis

The radiance collected from both diseased and healthy corals (L_s_) and the radiance from a white reference panel (L_r_) were utilized to calculate reflectance (R) at the same wavelength (λ) using the formula provided by Asner^19^:

$$R\left( \lambda \right)=\frac{{{L_s}\left( \lambda \right)}}{{{L_r}\left( \lambda \right)}}$$

A widely employed preprocessing technique in spectral analysis is the second derivative modification of reflectance spectra. This study identifies inflection points related to pigment absorption or structural changes, adjusts baseline shifts, and improves subtle spectral characteristics. This technique is particularly effective in coral reef research for identifying health-related changes in skeletal exposure and tissue pigmentation, often obscured in raw reflectance data.

The second derivative transformation was applied on the normalized reflectance spectra to improve the visibility of subtle absorption features while mitigating baseline shifts and noise. This method enhances the differentiation of overlapping pigment-related characteristics and increases sensitivity to spectral alterations induced by disease in corals. The second derivative was chosen over the first derivative due to its reduced sensitivity to random spectral noise and its superior capability in detecting minor variations linked to bacterial infection^20^.

The second derivative of the reflectance spectra was computed to enhance fine details in spectral signals, revealing subtle nuances not visible in the original curve and improving the differentiation between diseased and healthy corals. This approach facilitates the identification of unique patterns of peaks and troughs^21^.

The second derivative R′′(λ) of the reflectance spectra was computed employing the Savitzky-Golay filter (scipy.signal.savgol_filter, Python library: SciPy (v1.11.4)). An optimal window size and polynomial order were chosen to maintain spectral integrity, following the specified formula:

$$R^{\prime\prime}\left( \lambda \right)=\frac{{{d^2}R\left( \lambda \right)}}{{d{\lambda ^2}}}$$

Principal Component Analysis (PCA) was used using the scikit-learn module (v1.3.2) to diminish the dimensionality of the reflectance data while preserving the greatest variance^22^. PCA enabled the visualization of the categorization of healthy and sick coral reefs according to their spectral responses and supported further clustering analysis. The first two principal components (PCA1 and PCA2) were preserved and illustrated to depict the differentiation among groups.

### Bacterial isolation and identification

Coral fragments were placed in a sterile mortar, to which 5–10 mL of sterile seawater was added, followed by homogenization with a sterile pestle under sterile condition until the tissue was released into the liquid. Ten-fold serial dilutions were conducted on each sample from coral colonies using sterile marine salt water, and 100 µL of each dilution was applied to marine agar plates. All plates were incubated at 28 °C for 7 days, with daily observations to monitor bacterial growth. Morphologically different colonies were selected and re-streaked on new medium to produce pure isolates for further identification.

The bacterial isolates were identified by VITEK MS system. This technology facilitates rapid and precise identification of bacterial isolates, reducing dependence on conventional, laborious techniques. The VITEK MS system identifies bacteria by analyzing mass fingerprints using a sophisticated spectrum classifier algorithm, comparing the resulting spectra to a designated database, and doing identifications based on a single deposit without previous extraction from bacterial colonies^23^. Isolated bacterial colonies were deposited onto a target slide, covered with a matrix solution, and allowed to air dry. The VITEK MS system then evaluated the samples by matrix-assisted laser desorption ionization-time of flight mass spectrometry for identification purposes^24^. These bacterial profiles provide the microbial data necessary to explore potential spectral-microbial patterns distinguishing healthy and diseased coral colonies.

### Spectral-microbial data integration

All statistical analyses were conducted with Jupyter Notebook inside the Anaconda (Python v3.11) environment. The whole replicable pipeline included the following essential libraries and their respective versions: pandas v2.1.4, numpy v1.26.2, matplotlib v3.8.0, seaborn v0.12.2, scipy v1.11.4, scikit-learn v1.3.2, and scikit-bio v0.5.8.

### Unsupervised clustering approaches

a) Hierarchical clustering and heatmap visualization.

Hierarchical clustering was performed using the scipy.cluster.hierarchy module (v1.11.4) to evaluate the similarity of bacterial isolates based on their whole second-derivative spectral profiles^25^. A heatmap with dendrograms was generated using the seaborn library (v0.12.2) to visualize clustering patterns. The algorithm employs a distance metric (e.g., Euclidean distance, Manhattan distance) to quantify the spectral dissimilarity between every pair of bacterial samples. Smaller distances indicate higher spectral similarity. The method assumes that bacteria with similar biochemical compositions, cellular structures, or pigment profiles will exhibit similar light interaction properties (reflectance spectra). Cluster map generated to graphically depict grouping patterns and group-level distinctions.

b) K-Means clustering.

K-Means clustering, using scikit-learn (v1.3.2), was performed on the scaled spectral characteristics to categorize bacterial isolates into discrete groups. The ideal number of clusters (k) was established by the Elbow technique^26^, shown by graphing the inertia across a spectrum of k values (1–10). The clustering results were represented in PCA space for analysis.

### Canonical correspondence analysis (CCA)

Canonical Correspondence Analysis (CCA) was used to investigate the associations between bacterial spectrum patterns and environmental factors^27^, such as temperature, pH, and salinity. The analysis used scikit-bio (v0.5.8). The CCA biplot showed both the bacterial samples and the environmental gradients, emphasizing the influence of each element on spectral variance.

### Statistical analysis of environmental influence

To assess significant differences in environmental variables across the spectral clusters discovered by K-Means, both one-way Analysis of Variance (ANOVA) and the non-parametric Kruskal–Wallis were conducted based on the data distribution^28^. All environmental parameters (temperature, pH, salinity) were evaluated throughout the cluster groups. Boxplots were created to visually examine the distribution of environmental factors within each cluster.

## Results

### Bacterial community composition in healthy and diseased corals

**Fig. 4:**
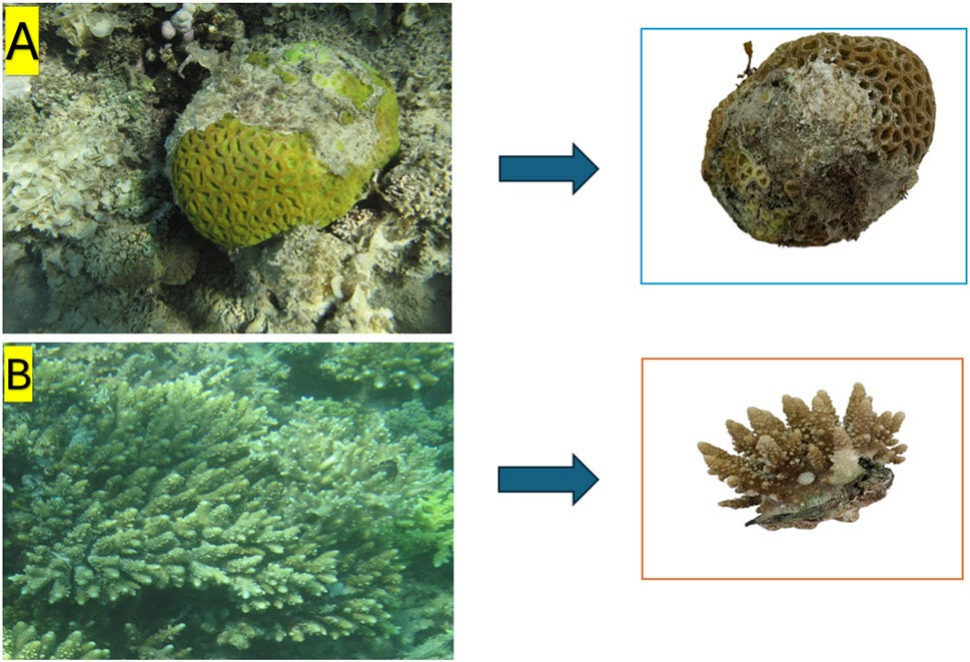
Photographs of the targeted coral colonies. **A** *Favia lacuna* exhibiting signs of White Plague Disease (WPD), showing a clear distinction between healthy, affected, and diseased tissue areas. **B** *Acropora humilis* displaying symptoms of White Band Disease (WBD).

All sample locations exhibited coral damage due to various diseases, particularly *Acropora* sp. and *Favia* sp., which were the most afflicted corals at both sites, all of which had identical coral reef architecture. The physicochemical properties of seawater at the sample locations are detailed in Table 1. The waters in the research region exhibited a temperature range of 16.84 to 17.9 °C, a pH range of 8.03 to 8.33, and a salinity of 36.9 to 37.15 PSU. Figure 4 illustrates the physical appearance of both healthy and diseased corals. *Acropora humilis* exhibiting disease signs consistent with white band disease exhibits symptoms characterized by the presence of a white band delineating healthy and necrotic coral tissues^29^. *Favia lacuna* showing visual characteristics resembling with white plaque disease exhibited accelerated tissue degradation and death^29^.

The structures of bacterial communities prevalent in healthy and infected corals were analyzed using MALDI-TOF mass spectrometry. Eight bacterial isolates were identified as the dominant bacteria in the selected coral colonies (Table 1). The isolates were grouped according to coral species and health status to improve clarity. In *Favia lacuna*, isolates *Bacillus subtilis* and *Bacillus amyloliquefaciens* were associated with healthy colonies, while isolates *Vibrio pelagius* were detected in diseased samples from both El-Shamaly and El-Shabrour reefs. In *Acropora humilis*, isolates *Cytobacillus firmus* and *Bacillus sporothermodurans* were obtained from healthy specimens, whereas *Vibrio fortis* was consistently associated with diseased corals across the studied sites. Overall, distinct differences in bacterial composition were observed between healthy and diseased coral tissues.

**Table 1:** Dominant bacterial isolates identified from healthy and diseased coral colonies across the two selected reef sites, along with the physicochemical characteristics of seawater at the sampling locations.

Coral species	Health status	Location	Dominant bacteria	Temperature	pH	Salinity (PSU)
*Favia lacuna*	Healthy	El-Shamaly reef	*Bacillus subtilis*	17.7 ± 0.081	8.18 ± 0.021	37.1 ± 0.22
	Healthy	El-Shabrour reef	*Bacillus amyloliqufaciens*	17.9 ± 0.047	8.29 ± 0.075	37.6 ± 0.16
	Diseased	El-Shamaly reef	*Vibrio pelagius*	17.7 ± 0.12	8.18 ± 0.025	37.1 ± 0.19
	Diseased	El-Shabrour reef	*Vibrio pelagius*	17.9 ± 0.15	8.29 ± 0.022	37.6 ± 0.14
*Acropora humilis*	Diseased	El-Shamaly reef	*Vibrio fortis*	16.84 ± 0.066	8.33 ± 0.024	37.15 ± 0.23
	Diseased	El-Shabrour reef	*Vibrio fortis*	16.88 ± 0.099	8.03 ± 0.022	36.9 ± 0.11
	Healthy	El-Shamaly reef	*Cytobacillus firmus*	16.84 ± 0.12	8.33 ± 0.026	37.15 ± 0.31
	Healthy	El-Shabrour reef	*Bacillus sporothermodurans*	16.88 ± 0.15	8.03 ± 0.032	36.9 ± 0.27

### Spectral differences between healthy and diseased corals

**Fig. 5:**
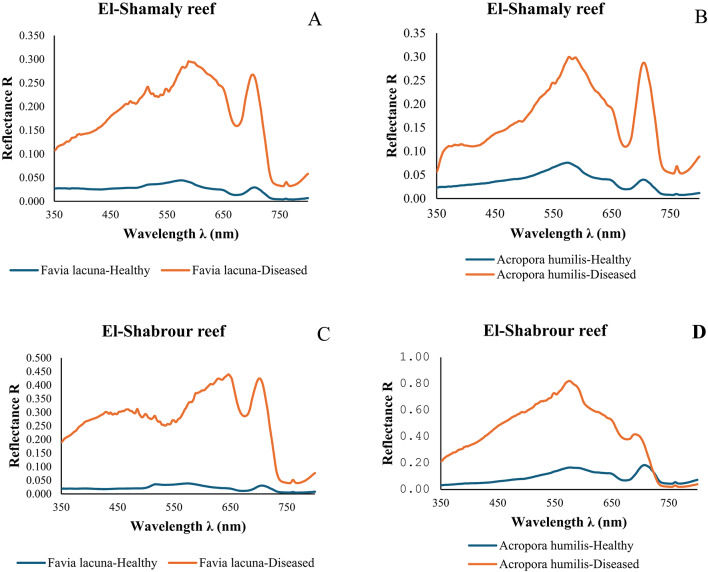
Mean spectral reflectance (R) characteristics of two healthy and diseased coral species across two reef locations, based on in-situ measurements. **A** Comparison between healthy and diseased *Favia lacuna* at El-Shamaly reef. **B** Comparison between healthy and diseased *Acropora humilis* at El-Shamaly reef. **C** Comparison between healthy and diseased *Favia lacuna* at El-Shabrour reef. **D** Comparison between healthy and diseased *Acropora humilis* at El-Shabrour reef.

One effective method for determining the distinctions between healthy and diseased corals is by applying spectral signatures. These methods show observable variations in the physical and biochemical characteristics of coral tissues, which serve as markers of health. There is a clear distinction between healthy and diseased coral colonies based on the spectral reflectance values in the visible to near-infrared spectrum (350–800 nm). Diseased samples exhibit noticeably higher reflectance, particularly in the visible (400–700 nm) and near-infrared (NIR: 750–800 nm) regions, while healthy samples show consistently lower reflectance across all bands. Spectral differences between healthy and diseased corals are evident in the visible light spectrum, particularly at 516 nm, 578 nm, and 700 nm bands, where raw curves display varying slopes, indicating differences in health status (Fig. 5).

Healthy *Favia lacuna* possesses spectral reflectance values between 0.004 and 0.044, demonstrating a slow rise in reflectivity from 500 to 572 nm, a decline after 708 nm, and a notable drop to 0.004 at 756 nm. In the case of diseased corals, the infected *Favia lacuna* exhibited a notable increase in reflectance, particularly at wavelengths of 594 nm, 649 nm, and 702 nm. The spectral pattern of diseased *Favia lacuna* is distinctly different from that of healthy corals, particularly in the Orange-red, Red, and Far-red (Red-edge) band (594–702 nm). This pattern serves as an effective indicator for differentiating between the two conditions through spectral analysis. The spectral reflectance profiles of *Acropora humilis* exhibit clear distinctions between diseased and healthy colonies, particularly within the visible spectrum (400–700 nm). Diseased samples display an abnormally high peak in the 580 nm and 702 nm bands. In accordance with the light absorption properties of symbiotic zooxanthellae, diseased samples have higher reflectance in all bands, particularly in the yellow-orange and Far-red regions (500–700 nm), while healthy samples continue to have lower reflectance in the blue and red bands.

### Second derivative spectral analysis for differentiating diseased and healthy coral colonies

**Fig. 6:**
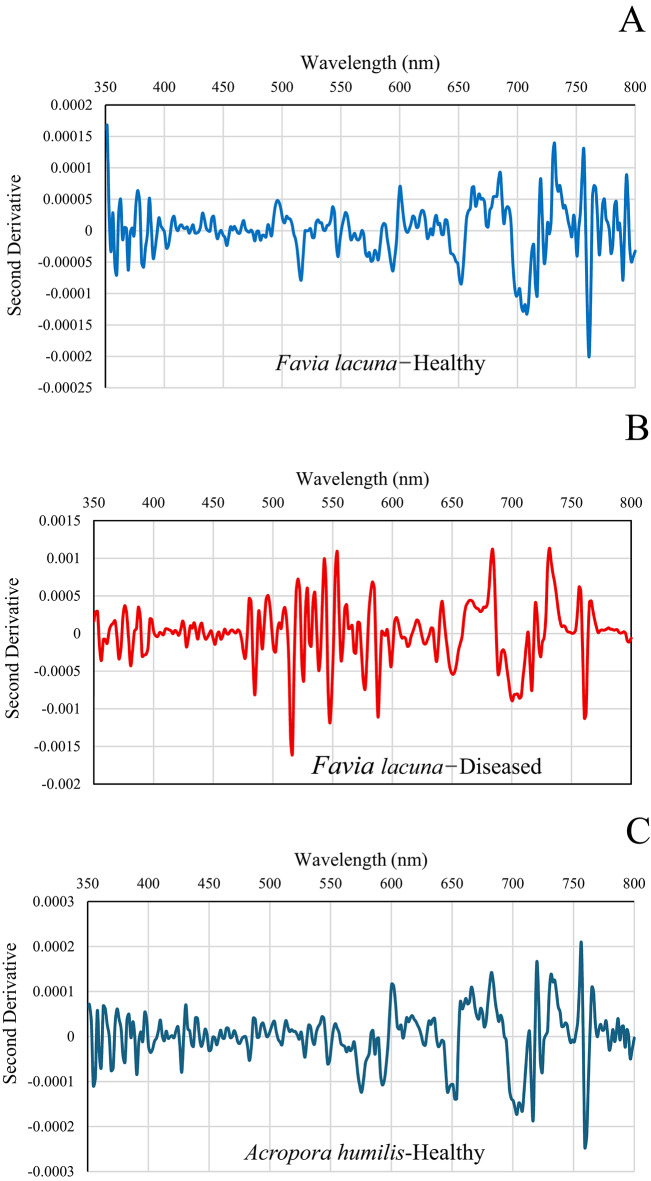

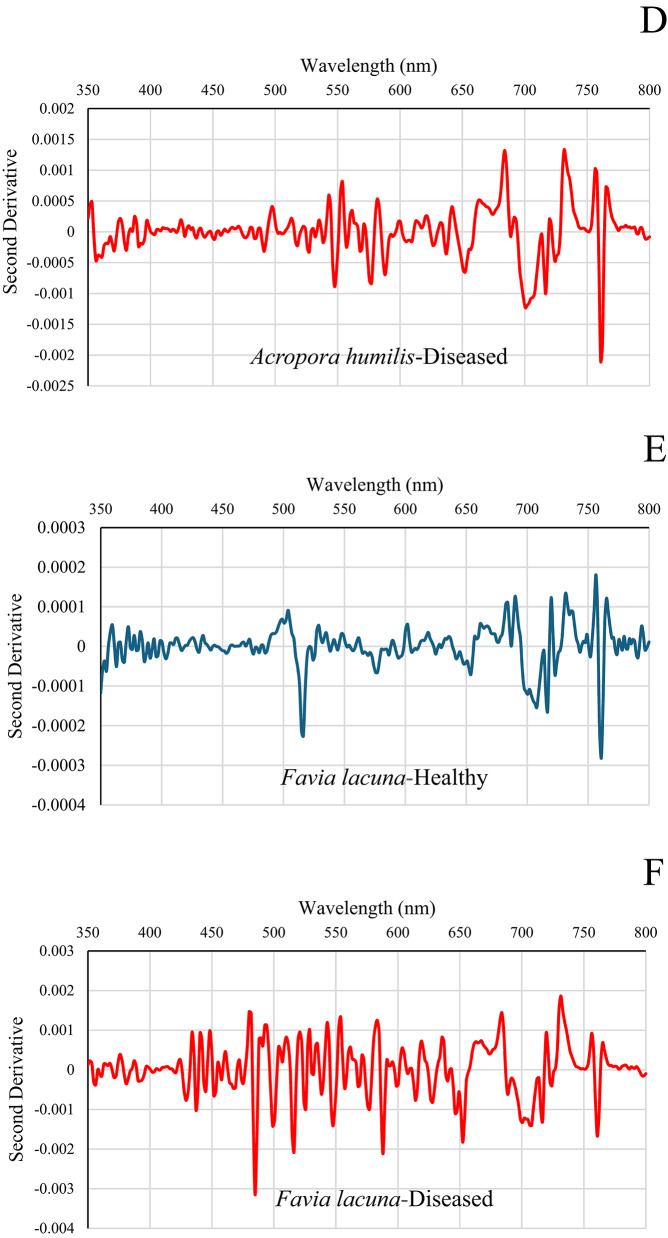

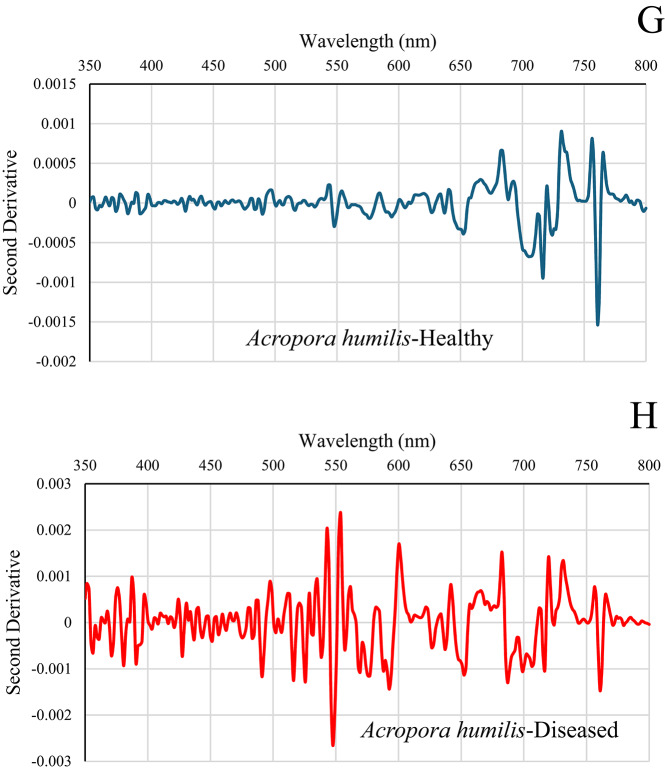
Second derivative spectral analysis of healthy and diseased coral colonies of *Favia lacuna* and *Acropora humilis* at two reef sites. **A**–**D** represent samples from El-Shamaly Reef, while panels **E**–**H** represent those from El-Shabrour Reef.

The second derivative spectral data for various coral species offers insights into their spectral characteristics across different wavelengths. The unique peaks and troughs of each coral colony display specific patterns in the second derivative spectra, which are associated with particular spectral features linked to their biological and physical characteristics. The second derivative analysis identifies wavelength regions of diagnostic significance, revealing clear and consistent differences between healthy and diseased colonies. Healthy *Favia lacuna* in El-Shamaly Reef (Fig. 6A) displayed second derivative curves that were overall smooth and symmetrical throughout the visible spectrum (400–700 nm). Localized negative peaks were identified at approximately 450 nm and 675 nm, aligning with the absorption characteristics of chlorophyll-*a* and photosynthetic pigments. These subtle troughs are characteristic of healthy coral reflectance, indicating pigment-related absorption overlaying a stable spectral baseline. The consistent spectral profile suggests intact symbiotic algal communities and minimal structural degradation. These sharp negative peaks suggest the presence of well-functioning photosynthetic pigments and stable tissue structures.

Diseased *Favia lacuna* in El-Shamaly Reef demonstrates broader and more variable second derivative curves in affected corals, characterized by diminished or displaced negative peaks, especially in the range of 670–700 nm, as illustrated in Fig. 6B. This suggests that chlorophyll-*a* has either degraded or been diminished, potentially due to tissue necrosis, or microbial overgrowth. Alterations in pigment composition and light-scattering properties due to tissue degradation or bacterial presence associated with disease are observable in specific spectra, characterized by the emergence of new negative peaks or the displacement of existing ones.

Healthy *Acropora humilis* in El-Shamaly Reef (Fig. 6C) displayed a spectrum with several distinct negative peaks, corresponding to specific absorption characteristics of coral-associated pigments and tissue structures. A significant trough at 675 nm is associated with chlorophyll-*a* absorption, which is characteristic of the photosynthesis process in symbiotic zooxanthellae. The peak is distinct and clearly delineated, signifying elevated pigment concentration and physiological integrity. Significant negative excursions were observed in diseased *Acropora humilis* at El-Shamaly Reef (Fig. 6D) at 580–590 nm, 649–653 nm, and 703–705 nm. The observed dips indicate a rise in absorption or a reduction in reflectance, typically associated with the structural breakdown of coral tissues, displacement of pigments, and degradation of chlorophyll-*a*. The magnitude and range of these negative peaks, particularly in the red and red-edge regions, signify considerable physiological stress. This stress may result from bacterial colonization or necrosis. A distinct negative peak is observed in the ~ 580–590 nm region (yellow range), which may indicate a decrease in carotenoid content or a modification in accessory pigments. This suggests stress-induced pigment breakdown, as carotenoids are known to decline under oxidative stress from microbial invasion or thermal anomalies, where a strong dip at approximately 650–653 nm (red range) is indicative of chlorophyll-*a* absorption, specifically the Qy band. The intensification of this peak in diseased corals indicates a decline in chlorophyll levels, potentially resulting from pathogenic activity that interferes with the photosynthetic apparatus or from the loss of symbiotic organisms (zooxanthellae). Notably, a pronounced negative peak in the range of approximately 703–705 nm, located within the red-edge region, serves as a potential diagnostic indicator. The concentration of pigment and the integrity of cellular structure both have an impact on this area. The decrease in diseased tissues indicates a disturbance in tissue organization and a reduction in pigment density, suggesting the presence of necrotic or bleached areas characterized by bacterial growth.

The distinct negative peak observed at around 675 nm in healthy *Favia lacuna* from El-Shabrour Reef is indicative of chlorophyll-*a* absorption, reflecting strong photosynthetic activity (Fig. 6E). Carotenoid/phycobilin accessory pigments are suggested by minor positive shoulders at 400–450 nm. Minimal non-photosynthetic interference is confirmed by baseline stability close to zero beyond 750 nm. A biomarker for intact photosynthetic apparatus is the deep negative peak at 675 nm, which is amplified by the second derivative. Asymmetric broadening of the 650–700 nm trough indicates metabolic stress, while elevated noise in the 500–600 nm range suggests breakdown of photopigments and cellular structures, and severe amplitude reduction at 675 nm indicates chlorophyll degradation in diseased *Favia lacuna* in El-Shabrour Reef (Fig. 6F). The diseased spectra’s 10x greater amplitude variation offers a measurable indicator of the severity of the pathology.

El-Shabrour Reef’s healthy *Acropora humilis* showed a dominant negative inflection at 675 nm, which is consistent with absorption of chlorophyll-*a* (Fig. 6G). Secondary features at 480 nm (carotenoids) and 530 nm (phycoerythrin) confirm pigment diversity. In the near-infrared range (greater than 700 nm), a flat baseline indicates minimal scattering artifacts. Decomposition improves the precision of health assessment by isolating signatures that are unique to a pigment.

The diseased *Acropora humilis* of El-Shabrour Reef demonstrates pathological changes in Fig. 6H, characterized by flattened derivative peaks across all wavelengths, suggesting a biochemical disturbance. Positive offset at 800 nm implies bacterial fluorescence or tissue necrosis. Incoherent noise in the 550–650 nm range masks typical pigment signatures; peak magnitudes indicate erratic scattering due to tissue degradation.

### Identification of spectral biomarkers for coral disease detection

The analysis of spectral reflectance data for healthy and diseased coral colonies of two species has identified consistent and diagnostically significant differences in specific spectral bands. The observed differences may function as spectral biomarkers—wavelengths that offer significant optical indicators of coral health status and facilitate disease detection in remote sensing applications. The band at 450 nm, located in the blue region, corresponds to the significant absorption of blue light by chlorophyll-*a* and various accessory pigments present in symbiotic zooxanthellae. Healthy corals show low reflectance in this region because of active pigment absorption, while diseased corals display increased reflectance, suggesting pigment degradation or loss. The rise in blue band reflectance (~ 450 nm) in diseased corals is typically linked to the loss of chlorophyll-*a* resulting from tissue degradation or the loss of symbionts, as documented in prior research^30^.

Diseased colonies exhibited elevated reflectance at the green band (500 nm), with *Acropora humilis* reaching a peak of 0.59. This observation indicates either significant tissue thinning or the presence of microbial or algal films on exposed skeletons. Healthy samples consistently exhibited low levels, thereby affirming the diagnostic potential of this range^31^. An observable and uniform increase in reflectance at 600 nm in diseased colonies indicates a significant reduction in pigment absorption and heightened light scattering due to tissue degradation or surface biofilms. Healthy corals, on the other hand, demonstrate significant amounts of absorption in this region, which leads to low reflectance values. Changes in internal structure or the exposure of the calcium carbonate skeleton are often indicated by the transition from red to near-infrared reflectance. In pathological samples, increased reflectance at 700 nm denotes tissue loss and structural deterioration. Low levels of reflectance are the result of healthy coral tissues primarily absorbing near-infrared light. Increased reflectance at 800 nm in diseased samples is a sign of dead or degenerated skeletons that strongly reflect in the near-infrared spectrum^32^. Table 2 presents the suggested spectral biomarkers.

**Table 2:** Key spectral biomarkers associated with coral disease detection. Summary of spectral bands showing distinct reflectance differences between healthy and diseased coral colonies. The biochemical relevance of each band is indicated, along with corresponding reflectance patterns and pathological interpretations. Values are based on in-situ spectral measurements.

Wavelength (nm)	Biochemical function	Reflection in healthy corals	Reflection in diseased corals	Indication
450 (Blue band)	Chlorophyll-*a* absorption	Very low (0.02–0.06)	High (up to 0.48)	Loss of pigments associated with disease
500 (Green band)	Green reflectance/bleaching signal	Low (0.04–0.14)	High (up to 0.95)	Tissue degradation or algal/microbial overgrowth
600 (Red band)	Red pigment absorption	Low (0.06)	Very high (up to 1.15)	Advanced pigment degradation, microbial colonization
700 (Red-edge band)	Skeleton exposure transition zone	Low (0.04)	High (up to 0.39)	Onset of tissue loss and skeletal visibility
800 (NIR band)	High reflectance from bare skeleton	Very Low (0.004–0.015)	High (0.04–0.08)	Severe tissue loss, indicative of necrosis or bleaching

### Principal component analysis (PCA)

Principal Component Analysis (PCA) was conducted on the second derivative spectral data of coral samples for assessing the efficacy of spectral patterns in distinguishing healthy from diseased coral colonies. The analysis decreased the dimensionality of the hyperspectral dataset, preserving the primary sources of variance related to coral health status. The initial two principal components (PC1 and PC2) represented a significant portion of the overall spectral variance. PC1 accounted for 53.45% of the variance, whereas PC2 accounted for 25.39%, together providing a thorough summary of spectral variability within the dataset. The 2D PCA scatter plot demonstrated a clear separation trend between healthy and diseased coral samples, with the majority of healthy colonies clustering in a specific region along the PC1 axis. This suggests that PC1 is significant in capturing health-related spectral differences.

**Fig. 7:**
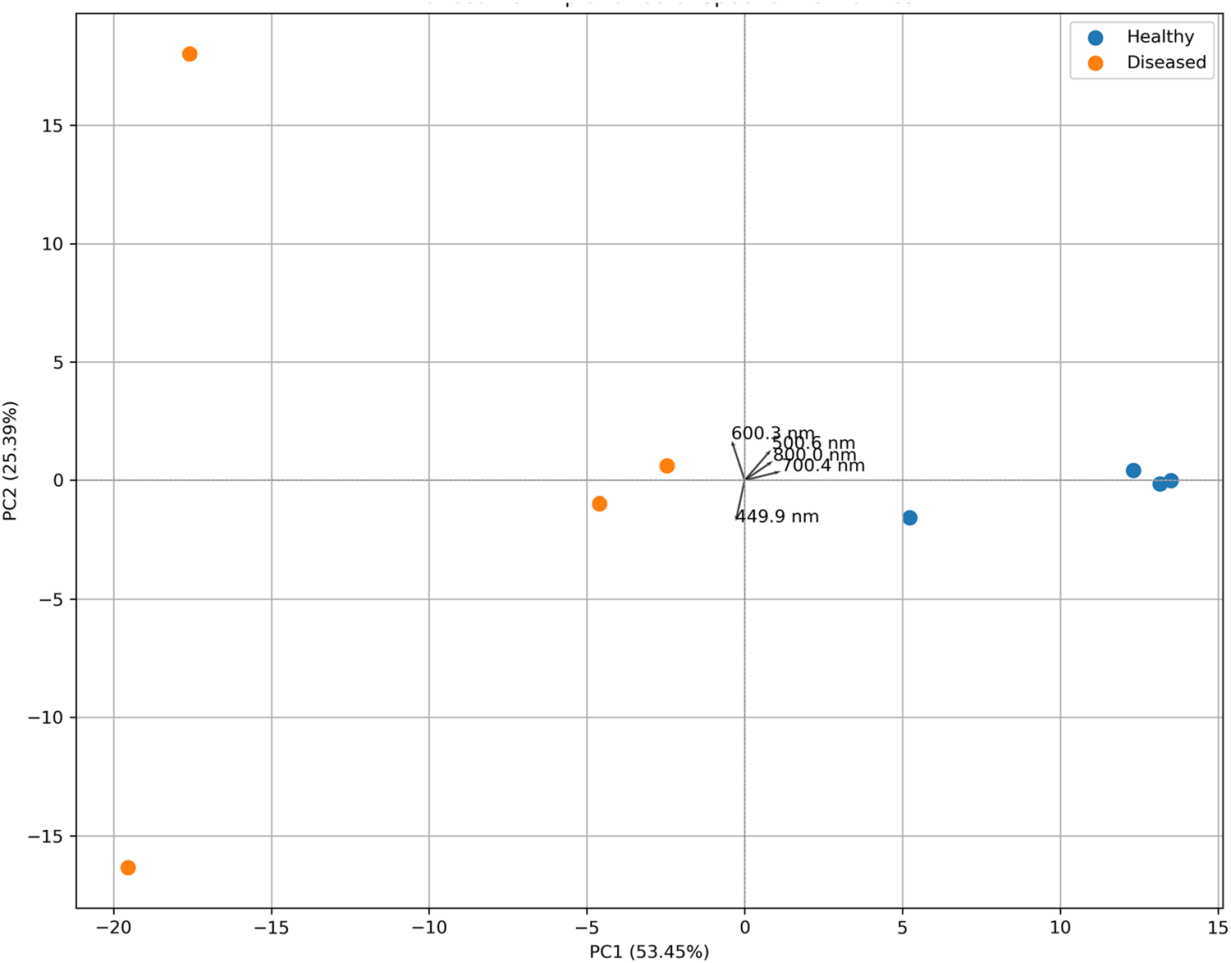
Principal Component Analysis (PCA) biplot based on second derivative spectral data of healthy and diseased coral colonies.

Independent t-tests were performed on the PCA scores to statistically validate this separation. Results indicated a significant difference in PC1 scores between healthy and diseased samples (t = 4.608, *p* = 0.00366), while PC2 did not demonstrate a statistically significant difference (t = − 0.093, *p* = 0.92864). This indicates that PC1 serves as the principal axis for differentiating coral health conditions according to their spectral profiles. The construction of a PCA biplot provided additional insights by overlaying the sample distribution with loading vectors that illustrate the contributions of individual wavelengths to PC1 and PC2. To minimize visual clutter and emphasize the most pertinent spectral features, only the ten wavelengths that significantly contribute to PC1 were represented as vectors (Fig. 7). The vectors identified specific spectral bands, notably at 500 nm, 600 nm, and 800 nm, as the most significant for distinguishing between the two health states. The orientation and magnitude of these vectors in the biplot demonstrate a significant correlation with the observed differentiation along PC1. The most prominent wavelengths observed indicate significant changes in the optical characteristics of coral tissue and/or the related microbial communities, making them promising spectral biomarkers for coral disease detection. The efficacy of hyperspectral second-derivative analysis in non-invasively differentiating coral health conditions is supported by the visual and statistical evidence from the PCA. Based on the absolute values of their loadings illustrated in Table 3, the top 10 wavelengths causing PC1 were numerically identified. The difference noted between the top 10 PC1 loadings and the biplot vectors arises from the contributions of both PC1 and PC2 in the biplot. As a result, specific wavelengths displaying moderate PC1 loadings in conjunction with strong PC2 loadings might be prominently featured in the biplot, although they do not rank among the top 10 contributors to PC1.

**Table 3:** Top ten wavelengths with the highest loading values contribute to the first principal component (PC1) derived from the second derivative spectral dataset of healthy and diseased coral colonies.

SN	Wavelength (nm)	Loading in PC1
1	650.8	0.0793
2	661.4	0.0791
3	665.9	0.0790
4	589.5	0.0789
5	659.9	0.0787
6	667.4	0.0786
7	730	0.0786
8	662.9	0.0784
9	664.4	0.0782
10	681	0.0775

### Linking spectral changes with bacterial infections

**Fig. 8:**
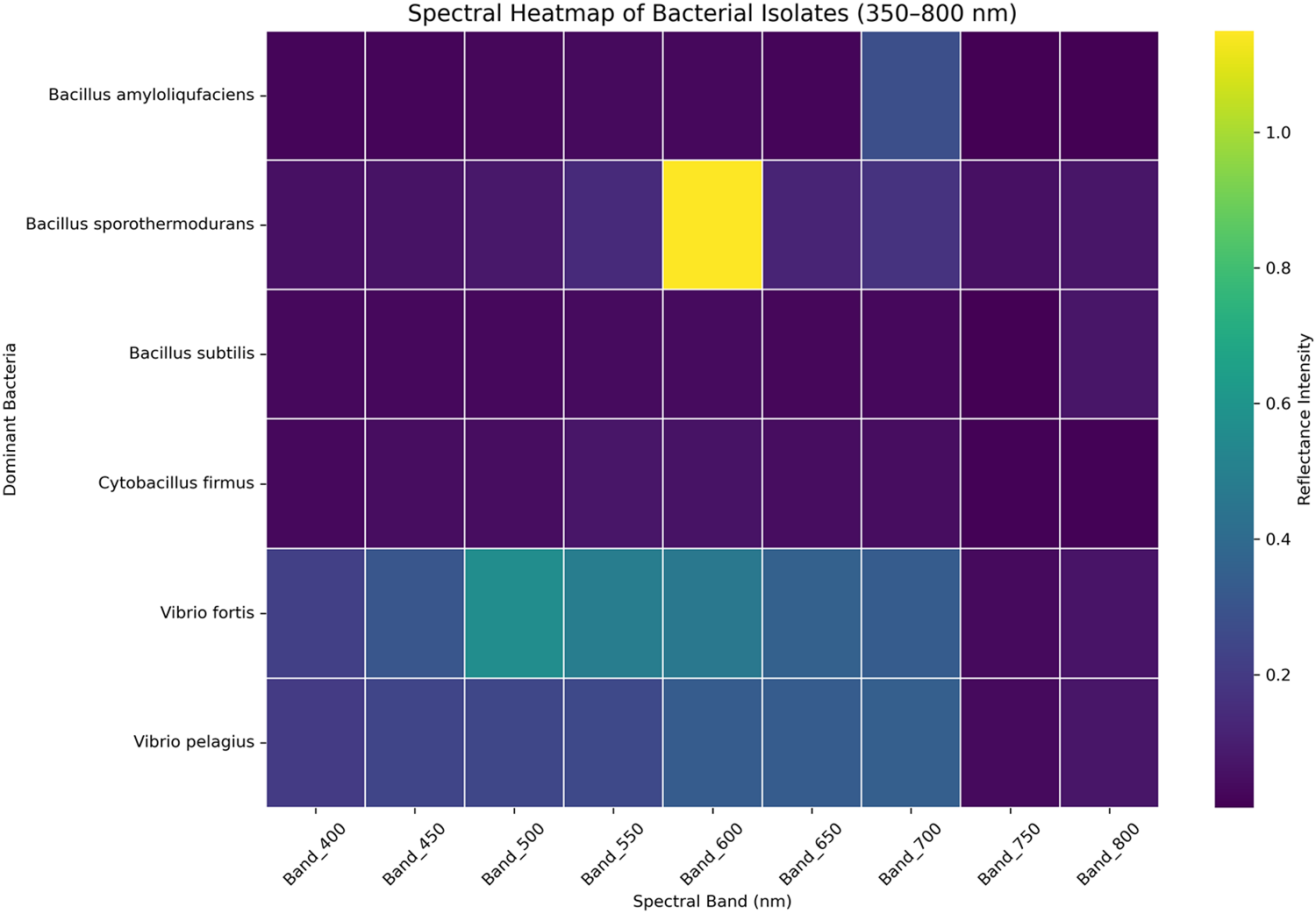
Spectral heatmap of dominant bacterial isolates (350–800 nm), displaying reflectance intensity patterns across the measured wavelength range. The heatmap reveals spectral variability among isolates, highlighting potential differences associated with bacterial identity or physiological state.

**Fig. 9:**
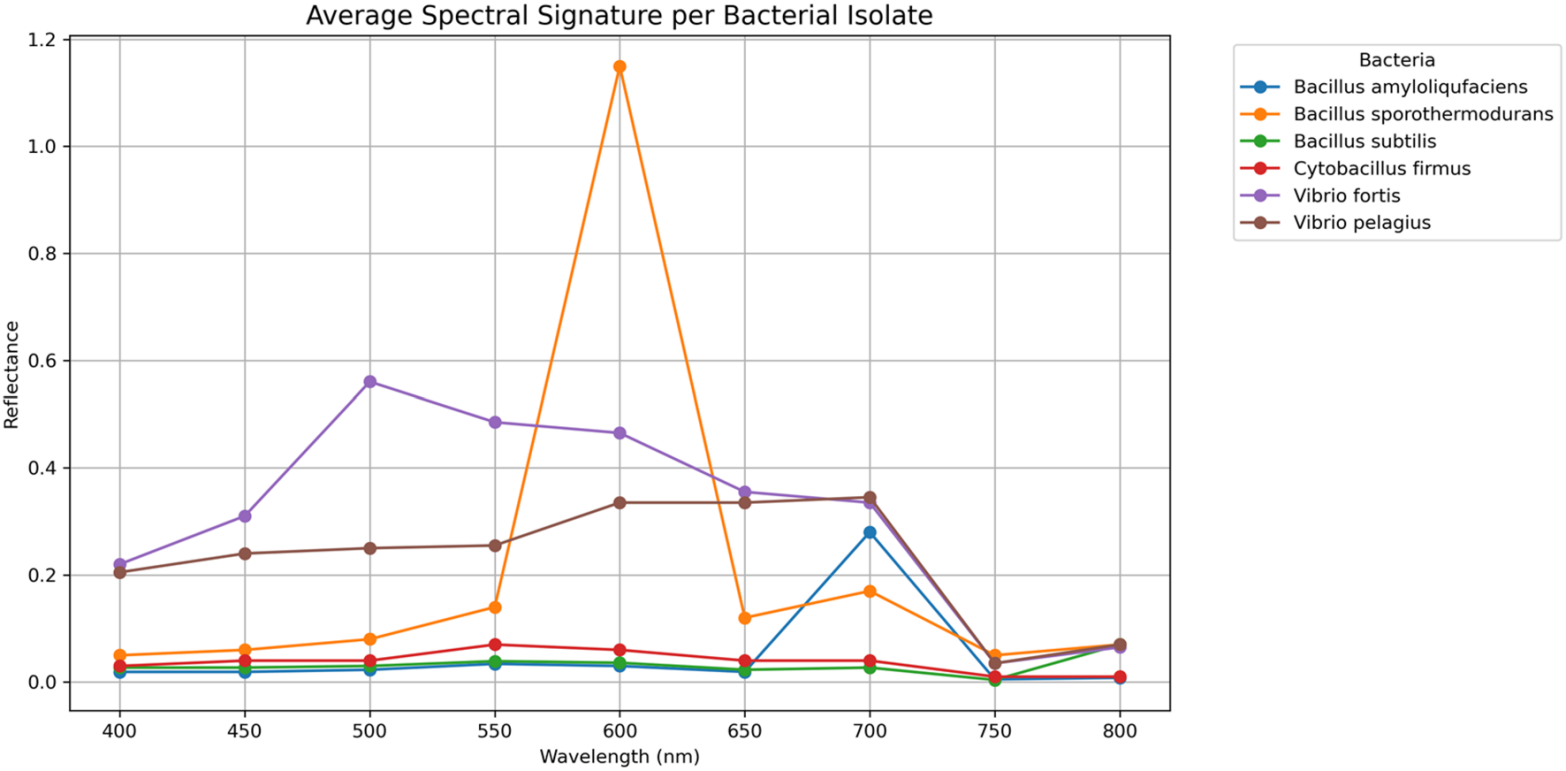
Spectral line plot showing the average reflectance signature for each dominant bacterial isolate. The plot highlights distinct spectral features that may differentiate between bacterial taxa or physiological conditions associated with coral health status.

Heatmaps and clustering were generated from raw spectral signatures for bacterial fingerprint analysis (Figs. 8, 9). The integration of spectral data with bacteria isolated from healthy and diseased coral colonies indicates that bacteria exhibiting high values at specific wavelengths suggested a potential interaction or signature within that spectrum. *Bacillus sporothermodurans*, isolated from healthy *Acropora humilis*, exhibits a high spectral reflectance of 1.15 at a wavelength of 600 nm.

The spectral signature of *Bacillus amyloliquefaciens*, which was isolated from healthy *Favia lacuna*, is 0.28 at 700 nm. The spectral signature of *Vibrio pelagius*, which was isolated from diseased *Favia lacuna*, is 0.35 at 750 nm. *Vibrio fortis* exhibits a high spectral reflectance of 0.56 at 500 nm after being isolated from diseased *Acropora humilis*. *Bacillus subtilis* and *Cytobacillus firmus*, isolated from healthy *Favia lacuna* and *Acropora humilis*, respectively, share a spectral signature of 0.07 at 800 nm and 550 nm.

**Fig. 10:**
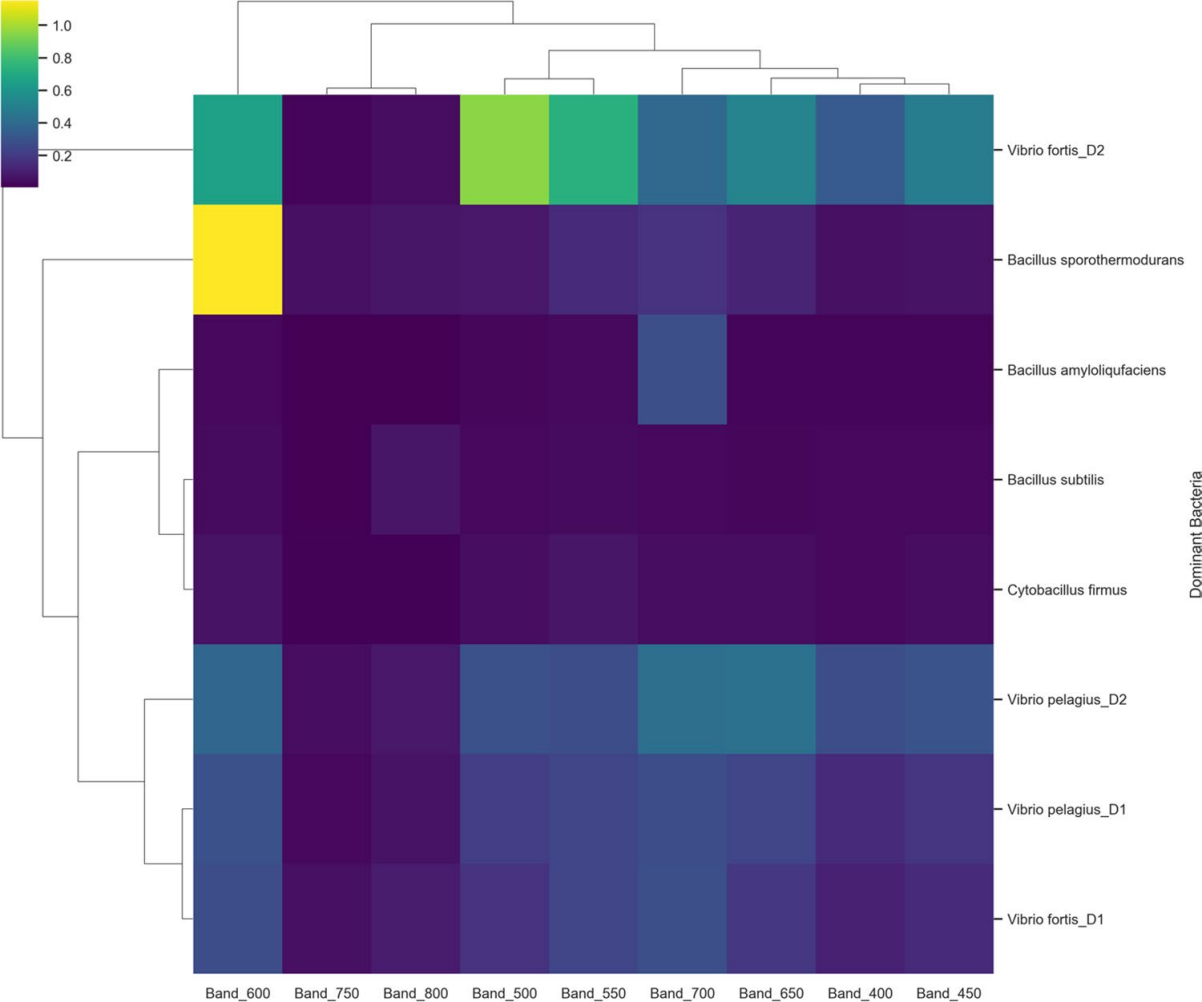
Hierarchical clustering heatmap with accompanying dendrogram illustrating the similarity in mean spectral reflectance among dominant bacterial taxa isolated from coral colonies. Clustering patterns reflect spectral affinities that may correspond to taxonomic or functional relationships.

The hierarchical clustering heatmap demonstrates similarity in mean spectral reflectance among dominant bacterial taxa derived from coral colonies. The reflectance values across bands from 400 to 800 nm are depicted using a color gradient, with the dominant bacteria in Fig. 10 organized according to their spectral similarity. The dendrogram analysis identified three primary spectral clusters, each comprising microbial samples with analogous reflectance characteristics, irrespective of the source health status.

Cluster 1, characterized by high near-infrared reflectance in the band range of 600–800, comprises *Vibrio fortis*-D2, isolated from site2, El-Shabrour reef, *Bacillus sporothermodurans*, and *Bacillus amyloliquefaciens*, all of which demonstrated increased near-infrared reflectance and significant peaks in the band range of 600–650. The spectral features are commonly linked to light-harvesting pigment systems present in symbiotic or phototrophic microbes, demonstrating low reflectance in most bands, especially within the visible spectrum. *Bacillus subtilis*, *Cytobacillus firmus*, and *Vibrio Pelagius*-D2(isolated from site2, El-Shabrour reef) were members of Cluster 2, which was identified by peaks in the 500–550 nm band. These bacteria showed notable reflectance in the green-visible spectrum (Band 500–550). This spectral behavior is frequently associated with oxidative stress adaptations or carotenoid content. Transitional or opportunistic microbial taxa present in both healthy and diseased coral colonies may be represented in the samples.

*Vibrio fortis*-D1 and *Vibrio Pelagius*-D1 which are isolated from El-Shamaly reef, are members of Cluster 3, which is distinguished by low reflectance in the 400–600 nm range. This group exhibits significantly reduced reflectance in the visible spectrum, especially within the 400–600 nm range. This cluster likely suggests the presence of bacteria that form biofilms or deplete pigments, which are linked to advanced stages of coral disease. The dendrogram branch lengths indicate the spectral distance between clusters, illustrating the efficacy of mean spectral signatures in differentiating bacterial communities linked to various coral health conditions. The heatmap’s color intensity corroborated these trends, indicating that disease-associated taxa, especially those in the red and near-infrared regions, exhibited higher reflectance values (e.g., yellow/green).

**Fig. 11:**
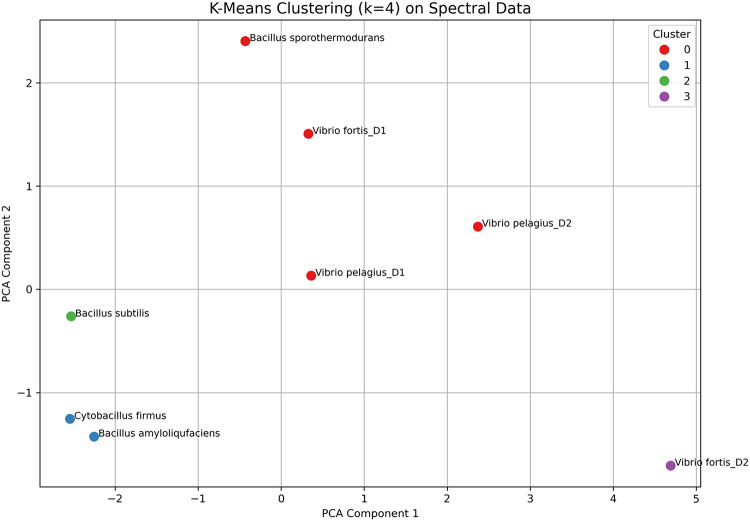
K-means clustering (k = 4) based on the mean spectral reflectance values across nine wavebands (400–800 nm), visualized in the PCA space for enhanced interpretation of group separation. Each bacterial isolate is colored by its assigned cluster and annotated according to its origin from either healthy or diseased coral colonies.

K-means clustering (k = 4) was applied to complement the hierarchical clustering results, as illustrated in Fig. 11, based on the mean spectral reflectance values across nine wavebands (400–800 nm). The clustering was represented in the PCA space, facilitating a clearer depiction of group separation. Each bacterial sample was categorized by its designated cluster and labeled with its health origin, indicating whether it was derived from healthy or diseased coral.

Cluster 0 includes *Vibrio fortis*-D1, *Vibrio Pelagius*-D1, *Vibrio Pelagius*-D2, and notably *Bacillus sporothermodurans*, indicating a convergence in their spectral profiles. The presence of a health-associated isolate (*Bacillus sporothermodurans*) within a primarily diseased group reflects the previous anomaly noted in hierarchical clustering, where spectral similarity surpasses health origin. This cluster exhibited moderate reflectance in Band (500–600), which may suggest the presence of transitional or stress-responsive bacterial taxa.

Cluster1 included *Cytobacillus firmus* and *Bacillus amyloliquefaciens*, demonstrating closely grouped positions in PCA space, which suggests significant spectral similarity. The profiles exhibited moderate reflectance across most bands, indicating a stable spectral signature potentially associated with symbiotic or core members of the healthy coral microbiome. Cluster 2, consisting solely of *Bacillus subtilis*, exhibited distinct characteristics compared to the other clusters. The bacterial sample had a spectral signature that was unique compared to both healthy and non-healthy samples. The reflectance features suggest it may belong to a functionally specialized or ecologically unique group. The reflectance features suggest it may belong to a functionally specialized or ecologically unique group. Cluster 3 consisted only of *Vibrio fortis*-D2, which demonstrated a marked distinction from the other clusters, consistent with its previously identified anomalous placement in the dendrogram. The increased reflectance in the 750–800 nm range indicates unique phototrophic traits, notwithstanding its pathogenic attributes. This reinforces the prior conclusion that optical characteristics alone may not always indicate pathogenicity without further functional context.

A Kruskal–Wallis H-test was conducted to evaluate the influence of environmental gradients on the identified spectral bacterial clusters by comparing temperature, salinity, and pH levels across the four clusters. The findings showed no statistically significant differences among clusters for the measured environmental variables, including temperature (H = 0.312, *p* > 0.05), salinity (H = 0.589, *p* > 0.05), and pH (H = 1.29, *p* > 0.05). The results reveal that changes in environmental factors haven’t got a significant impact regarding how bacterial isolates are grouped based on their spectral signatures. The differences in the spectra that were seen can be explained by the microbes’ own characteristics, such as their pigment composition, metabolic state, or stress response mechanisms, without considering any outside abiotic factors. This demonstrates the effectiveness of the spectral method in distinguishing bacterial functional groups and health-state associations, even in the presence of moderate variations in temperature, salinity, or pH.

**Fig. 12:**
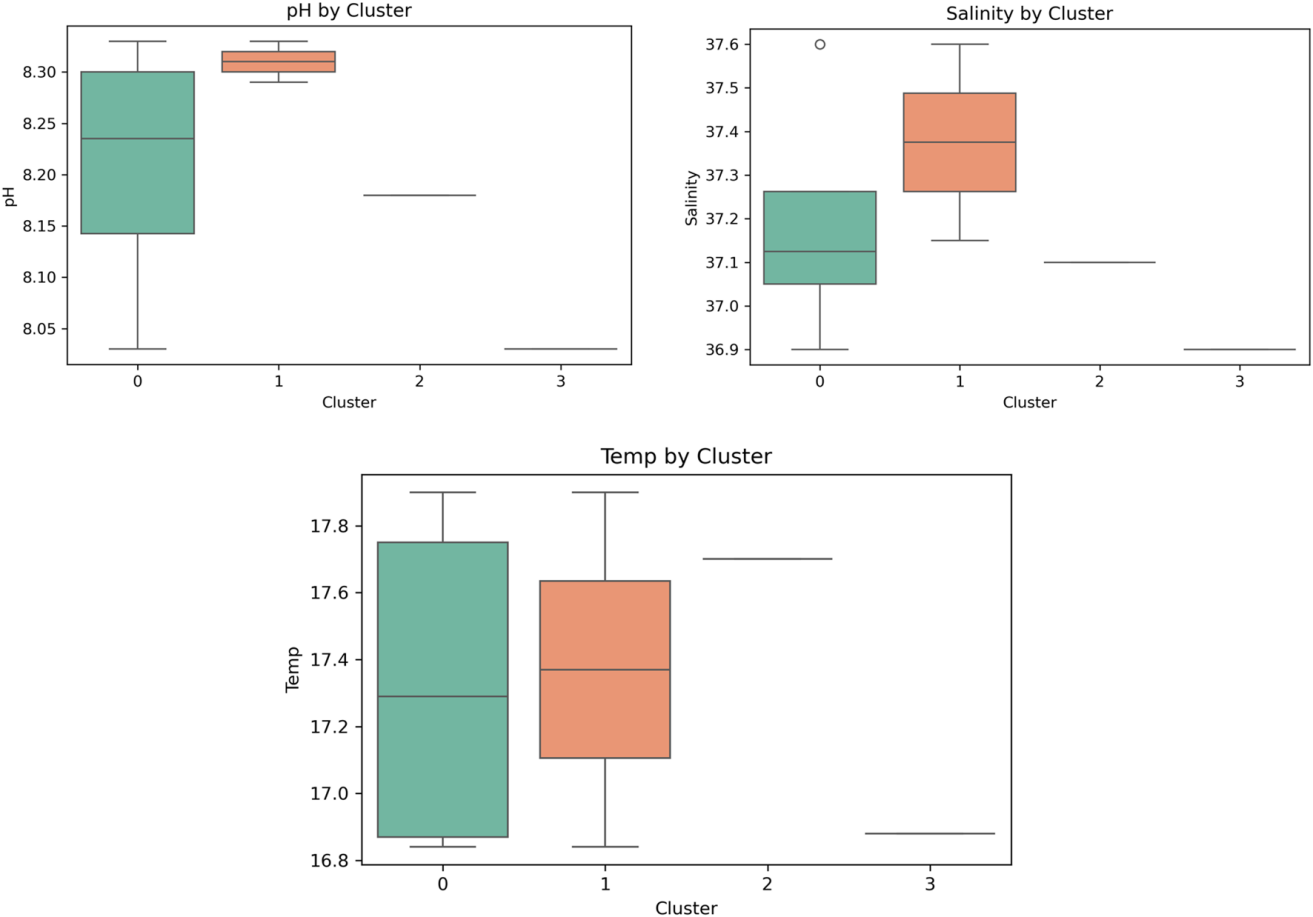
Boxplots illustrate the distribution of environmental parameters (temperature, salinity, and pH) across the four spectral clusters derived from K-means analysis. The Kruskal-Walli’s test was applied to assess statistically significant differences among clusters.

Descriptive statistical studies were conducted on the four spectral clusters to investigate the correlations between environmental conditions and bacterial cluster identities, specifically focusing on temperature, salinity, and pH (Table 4). Boxplots were created to illustrate the distribution of each variable across clusters (Fig. 12). Mean temperatures among clusters exhibited little variance, ranging from 16.88 to 17.7 °C. Cluster 2 had a somewhat elevated median temperature compared to the others; nevertheless, the interquartile ranges displayed substantial overlap, as seen by the non-significant findings of the Kruskal–Walli’s test. All clusters exhibited similar salinity ranges (36.9–37.75 PSU), with Cluster 0 demonstrating the least fluctuation. This homogeneity suggests that salinity was comparatively consistent across sample locations and unlikely to affect bacterial spectrum grouping. The pH values consistently fell within a limited range (8.03–8.31), with little variations in medians across clusters. Cluster 1 had the least pH change, suggested potential stable environmental conditions within this group. In contrast, larger variance was detected in Cluster 0, but discrepancies remained within normal marine pH limits.

**Fig. 13:**
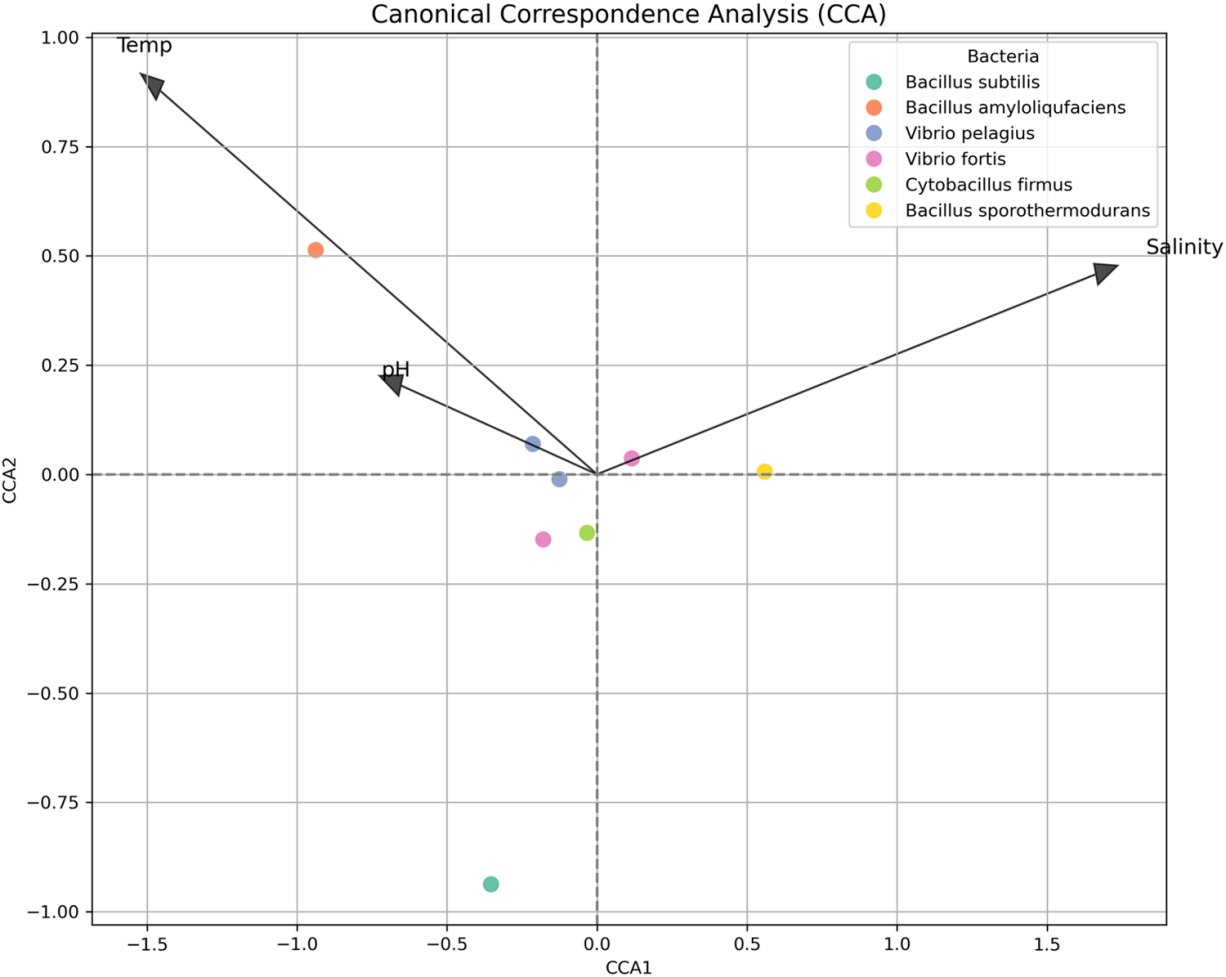
Canonical Correspondence Analysis (CCA) biplot illustrating the relationships between dominant bacterial taxa and environmental variables (temperature, salinity, and pH). Arrows represent the direction and strength of environmental gradients, while points indicate the position of bacterial taxa in relation to these gradients.

The relationship between bacterial taxa and environmental factors including pH, salinity, and temperature was investigated using canonical correspondence analysis (CCA). The spatial distribution of the different bacterial isolates along the primary canonical axes in the CCA biplot (Fig. 13) revealed relatively narrow ranges. *Bacillus sporothermodurans* was situated in close alignment with the salinity vector at the positive end of the CCA1 axis. This suggests that there is potential association with saline conditions. *Bacillus amyloliquefaciens* showed an apparent alignment with the temperature vector. Given the narrow temperature range observed in this study, this pattern is more likely to reflect minor environmental variation rather than a definitive physiological preference, particularly as this species is generally considered mesophilic. Positioned in the lower-left quadrant of the biplot, *Bacillus subtilis* revealed a notable separation from all environmental vectors, suggesting that it may be environmentally intolerant or prefer conditions that are not represented by the variables being measured.

*Vibrio pelagius*, *Vibrio fortis*, and *Cytobacillus firmus* were among the bacterial species that were grouped around the origin, suggesting a weak to moderate response to the environmental gradients. These limited variations suggest that the observed CCA patterns are more likely influenced by subtle spatial or micro-environmental differences rather than strong environmental gradients. Therefore, the relationships between bacterial distribution and environmental variables should be interpreted with caution. Therefore, the environmental vectors’ orientation and length likely reflect that temperature and salinity were the main influencing factors, while pH had a less pronounced but still noticeable impact. The findings reveal that environmental conditions may impact the makeup of the bacterial population associated with coral colonies. In reef systems subjected to salinity or thermal stress, certain species may act as markers of environmental stress; however, these patterns need to be evaluated with caution because of the restricted sample size and statistical significance.

**Table 4:** Descriptive statistics of environmental parameters (temperature, salinity, and pH) across the four spectral clusters. Mean and standard deviation values are presented for each parameter.

Cluster	Temperature		pH		Salinity	
	Mean	Std	Mean	Std	Mean	Std
0	17.33	0.55	8.21	0.13	37.19	0.30
1	17.37	0.75	8.31	0.03	37.38	0.32
2	17.7	N/A	8.18	N/A	37.1	N/A
3	16.88	N/A	8.03	N/A	36.9	N/A

Standard deviation was not calculated for clusters containing only one sample (e.g., Cluster 2 and Cluster 3).

## Discussion

### Overview of spectral and microbial integration

This study employed an underwater hyperspectral spectroradiometer and microbial profiling to characterize coral reef health across a wavelength range of 350–800 nm resampled at 1 nm intervals. This spectral interval aligns with underwater light attenuation characteristics and previous coral optical studies^33^. The results demonstrate that the spectral signatures can reliably differentiate between healthy and diseased coral colonies and that these optical differences correspond with shifts in microbial community composition. Unlike prior research that has relied mainly on characterizing coral disease via macroscopic symptoms, histological evaluations, or metagenomic sequencing, our study provides a nondestructive methodology integrating reflectance spectroscopy with microbial profiling^34^. The derivative spectral signature and multivariate clustering support the concept of microbial fingerprints as a rapid diagnostic indicator of coral reef health.

### Microbial community shifts and pathogenic bacteria linked to coral health status

Bacteriological analysis revealed a clear dichotomy between the microbial communities of healthy and diseased corals. Healthy colonies were dominated by mutualistic taxa known to support nutrient cycling and holobiont stability, whereas diseased samples showed increased representation of opportunistic and pathogenic taxa, consistent with microbial dysbiosis patterns documented in stressed coral^34–36^. The identification of potentially pathogenic or stress-tolerant bacteria in diseased samples corresponds with the “Anna Karenina Principle” in coral microbiomes, indicating that microbial variability escalates in response to host stress^37^. The results highlight the significance of microbial changes as preliminary signs of declining coral health. Although the number of bacterial isolates analyzed (*n* = 8) is limited, the observed patterns provide proof of concept that spectral microbial linkages are detectable in situ. The expanded future sampling will be required to validate ecological consistency across sites and seasons.

The microbial profiling in this study demonstrated a distinct separation between bacteria linked to healthy and diseased coral colonies, consistent with patterns identified in prior coral disease research. In *Favia* corals impacted by white plague disease, *Thalassotalea loyana* was identified as a significant pathogen, aligning with prior isolations from the Red Sea^38,39^. In our study, *Vibrio pelagius* is regarded as the primary pathogen responsible for White Plague disease in *Favia lacuna*. The bacteria implicated in white band disease (WBD) affecting *Acropora* corals in the Red Sea have been identified through multiple studies, demonstrating a complex interaction of microbial communities. Studies demonstrate that WBD correlates with particular pathogenic bacteria, notably from the *Vibrio* genus and Rickettsiales-like organisms, which are associated with the deterioration of coral health. Research has identified *Vibrio alginolyticus* and *Vibrio owensii* as notable pathogens linked to WBD in *Acropora*spp^40^. Our research identifies *Vibrio fortis* as the primary pathogen responsible for WBD in *Acropora humilis*.

Bacterial isolates from healthy corals in both genera mainly encompass commensal or mutualistic taxa, including phototroph-associated lineages. These lineages exhibited much greater NIR reflectance and did not demonstrate any harmful characteristics in culture-based tests. Based on the patterns, it seems that bacteria that relate to diseases often have unique optical and taxonomic fingerprints in comparison to microbiota that are generally associated with health. The spectral analyses revealed differences, with pathogenic taxa clustering within low-reflectance or structurally distinct spectral groups as identified in the Hierarchical Clustering Analysis (HCA) (e.g., Cluster 3) (Fig. 10). This finding is consistent with research in environmental microbiology, which shows that disease-driving bacteria can modify community structure and biochemical composition, subsequently affecting optical properties^41^.

### Spectral variation between healthy and diseased coral colonies

This research analyzes the spectrum variations between healthy and diseased corals, highlighting observable optical variances in mean reflectance spectra linked to bacteria associated with corals. The near-infrared (NIR) and visible red bands exhibited consistently elevated reflectance in healthy samples, likely attributable to pigmented phototrophic bacteria. Conversely, disease-associated bacteria exhibited reduced reflectance in most bands, particularly in the visible blue (400–500 nm) and green (500–570 nm) regions. Consistent with prior research emphasizing the sensitivity of hyperspectral signatures to microbial and physiological alterations in corals, the noted spectral variations may reflect modifications in bacterial cell wall composition, pigment concentration, and metabolic condition^42,43^. The potential of reflectance as a swift indicator for evaluating microbial health is shown by significant differences in spectral patterns.

This study employs second derivative spectral transformation, comparable with the methods used by Clark et al.^44^ effectively identifying inflection points and mitigating baseline changes to improve small variations in reflectance profiles. In comparison to raw spectra, this approach showed improved spectral features in diagnostic bands, increasing the sensitivity of health state discrimination. The improvement allows for more precise separation of wavelength-specific changes linked to bacterial pigmentation and structural changes in diseased samples, which is consistent with well-established uses of derivative spectroscopy in remote sensing for differentiating target compounds in complex environments.

### Second derivative spectral biomarkers and biological interpretation

The second derivative reflectance profiles revealed genus-specific and infection-related spectral anomalies in both Favia and Acropora, supporting earlier findings by Joyce and Phinn^30^, and Kutser et al.^32^, who showed that second derivative analysis improves the identification of subtle spectral features associated with coral pigmentation, tissue structure, and skeletal morphology. Significant absorption characteristics were noted in diseased *Favia* and *Acropora* colonies, especially at wavelengths of 450–460 nm, 580–590 nm, 675 nm, 700–740 nm, and 780–800 nm. These spectral anomalies may suggest an increased presence of microbial biofilms or the degradation of symbiotic pigments, which relates to pathogen-associated second derivative spectral features. For instance, the presence of a distinct alteration in chromogenic bacteria or the presence of exogenous pigments, such as prodigiosin or pyocyanin^45^, is observed at a wavelength of 450 nm. The range of 580–600 nm suggests the presence of bacterial pigments or tissue degradation, while a reduction in reflectance at 675 nm in the chlorophyll absorption peak may indicate the destruction of zooxanthellae due to bacterial infection^46^. Indicators of bacterial activity resulting from the degradation of surface tissues are observed at wavelengths of 700–740 nm^42^. Elevated reflectance observed at 780–800 nm in infected corals may be associated with advanced tissue degradation and microbial colonization^32^.

Our study expands upon the research conducted by Khaled and Abdelsalam^47^, who used second derivative hyperspectral analysis to discriminate coral taxa but did not incorporate disease state. This study focused exclusively on the taxonomic differentiation among healthy colonies of *Acropora*, *Porites*, and *Pocillopora*. Our study expands the viewpoint by incorporating health status as a discriminant factor, particularly concerning bacterial coral diseases. Khaled and Abdelsalam’s results did not capture these features, likely due to their narrow focus on unaffected specimens. Furthermore, within the same species, our PCA was able to separate samples based on the status of infection, indicating that microbially induced changes may be just as spectrally significant as interspecific variation. This illustrates the potential of hyperspectral sensing for species-level classification and near real-time coral health monitoring.

### Multivariate analysis and diagnostic spectral features

Principal Component Analysis (PCA) successfully reduced the dimensionality of second-derivative hyperspectral reflectance data, facilitating the extraction of predominant spectral patterns and reducing noise. The initial two principal components (PC1 and PC2) collectively represented a significant portion of the total variance, with PC1 predominantly reflecting the spectral disparities between healthy and diseased coral colonies. Reflectance variations across the 550–800 nm spectrum, linked to microbial biofilms, symbiont loss, and chlorophyll degradation^48,49^, had a significant impact on the axis. Although accounting for a lesser proportion of the variance, PC2 seems to represent intra-group variation, possibly associated with differences in secondary pigmentation or the composition of the bacterial population.

The PCA biplot corroborated these results, indicating that the top 10 wavelengths exhibiting the highest loadings were in close alignment with the previously identified diagnostic spectral biomarkers, specifically the peaks observed around 560, 650, and 750 nm. The spectral divergence between the groups was likely caused by pigmented microbial assemblages, which may have included photosynthetic symbionts or pigmented pathogens. A t-test on PC1 scores indicated a statistically significant difference (*p* < 0.05) between healthy and sick coral samples. The results demonstrate that PCA serves as a dependable technique for the extraction of physiologically and diagnostically relevant information from coral spectral data, particularly when integrated with microbiological characterizations.

Loading scores obtained from PCA were used to identify spectral biomarkers, and the results revealed that certain wavelengths, specifically those around 590 nm, 650 nm, and 730 nm were important in distinguishing between healthy and diseased groups. The bands represent regions where vital microbial pigments, such as phycobiliproteins, carotenoids, and chlorophyll, are absorbed. These pigments are often altered in microbial communities that are under stress or diseases. The capacity of these bands to differentiate among various health states aligns with previous attempts to identify hyperspectral disease indicators in coral tissues^30,50,51^. Thus, specific spectral bands may function as non-invasive biomarkers for disease surveillance and early identification.

The integration of hyperspectral reflectance data with microbial community profiles indicates that specific spectral changes are significantly linked to functional differences in bacterial composition. Elevated near-infrared (NIR) reflectance, specifically within the 700–800 nm range, suggests a predominance of phototrophic symbionts, including photosynthetic bacteria and endosymbiotic dinoflagellates, in healthy coral colonies. Diseased colonies showed significant reductions in reflectance within the visible spectrum (400–600 nm), indicating the proliferation of non-pigmented, pathogenic taxa, such as biofilm-forming heterotrophs. The optical fingerprints coordinate with known physiological changes linked to microbial dysbiosis, including microbial overgrowth, mucosal layer disruption, and pigment degradation^52–54^.

Hyperspectral sensing combined with cluster analysis effectively distinguishes between the spectral signatures of healthy and diseased coral, facilitating prompt identification of coral disease outbreaks^10^. Spectral clustering techniques, such as Hierarchical Clustering Analysis (HCA) and K-means, effectively classified bacterial isolates into ecologically and functionally relevant groups that closely aligned with coral health status. HCA identified three principal spectral clusters: cluster 1, characterized by elevated near-infrared (NIR) reflectance, typically associated with phototrophic communities; cluster 2, which exhibits a peak in the green-visible range (~ 500–550 nm), linked to opportunistic or stress-adapted taxa; and cluster 3, distinguished by low reflectance across the visible spectrum, indicative of pathogenic or pigment-deficient taxa.

The K-means method independently corroborated this structure, augmenting the repeatability of the spectral divisions. The clustering primarily corresponded with microbial function and the health status of coral, with some notable exceptions. The spectral characteristics of *Vibrio fortis*-D2, identified as a pathogenic isolate, exhibited similarities to those of healthy samples and indicated evidence of phototrophic growth. Conversely, disease-associated bacteria were found to be concentrated in *Bacillus sporothermodurans*, which was initially obtained from a healthy colony. Research on coral microbiomes reveals that the observed anomalies highlight the complexity of microbial communities and may suggest context-dependent spectral behavior or transitional metabolic states^53^.

The clustering of samples, including *Bacillus amyloliquefaciens*, *Cytobacillus firmus*, and *Bacillus subtilis*, which exhibited close grouping in both studies and are linked to healthy coral, illustrates the alignment between K-means and hierarchical clustering methodologies. The spectral separation of *Vibrio fortis*-D2 in both clustering approaches confirms its classification as an outlier, thereby improving its unique spectral identification. The mismatch of *Bacillus sporothermodurans* with primarily diseased samples underscores the limitations of unsupervised grouping based exclusively on reflectance, as well as the potential for optical signal overlap among different health statuses. The assertion that spectral reflectance can differentiate between microbial populations of functional and ecological significance is substantiated by the integration of K-means and hierarchical clustering methodologies. Nevertheless, some overlap persists, necessitating additional correlation with phenotypic or genomic data to confirm the validity of the clusters.

### Environmental parameters and spectral grouping

A non-significant difference in temperature, salinity, or pH was detected among bacterial optical clusters (Kruskal–Wallis, *p* > 0.05). This suggests that the observed spectral segregation is influenced by microbial characteristics, such as pigment composition and cellular morphology, rather than by abiotic gradients. Canonical Correspondence Analysis (CCA) supported this observation, showing weak to negligible correlations between cluster positions and environmental vectors. The suggestion is that the observed spectral patterns are likely related to bacterial identity and function, rather than influenced by local environmental fluctuation.

The integration of hyperspectral sensing, derivative spectral diagnostics, and microbial profiling establishes a scalable and non-destructive framework for coral health evaluation. Consistent with recent machine learning and remote sensing studies applied to reef monitoring^54^. our findings demonstrate that microbial spectral biomarkers can serve as early indicators of dysbiosis and disease progression.

## Limitations of the study and future recommendations

This research includes limitations that must be recognized when evaluating the results. The limited number of bacterial isolates examined diminishes statistical power and restricts the generalizability of the microbial-spectral relationships identified in this study, and it is important to note that disease identification was based on visual field observations, and no molecular or histopathological confirmation was performed. Therefore, disease classifications should be interpreted with caution, particularly for conditions with overlapping clinical signs.

The research concentrated only on two coral taxa, perhaps overlooking the wider taxonomic diversity in disease responses across Red Sea reef ecosystems. The results herein should be regarded as a proof-of-concept framework demonstrating the potential of integrating microbial profiling with hyperspectral analysis, while underscoring the necessity for larger datasets, broader taxonomic coverage, and in-situ spectral validation in subsequent research. Future studies should (i) validate the identified spectral biomarkers through metagenomic or meta-transcriptomic data, (ii) broaden the spectral data library to encompass a greater diversity of coral-associated microbes across various seasons and reef regions, and (iii) combine in situ spectral measurements with drone or satellite platforms to monitor coral reef health in real time, thereby improving the potential diagnostic effectiveness of hyperspectral microbial analysis. The application of machine learning models to spectral datasets can enhance classification accuracy and enable early detection of bacterial imbalances or disease outbreaks prior to the manifestation of noticeable symptoms. The implementation of those efforts will highlight the value of microbial spectroscopic tools in the preservation of coral reefs, especially in regions like the Red Sea that are vulnerable to climate change.

## Conclusion

This research presents an exploratory framework that combines microbiological profiling with hyperspectral reflectance analysis to identify spectral and microbial variations between healthy and diseased coral colonies in the Red Sea. The findings, though limited by the small number of bacterial isolates, offer preliminary insights and demonstrate the potential of integrating spectral and microbial methods for future extensive assessments of coral health.

To validate the practical applicability of our approach for field-level disease detection, we identified the spectral wavebands that reliably distinguished healthy from diseased *Acropora* and *Favia* colonies in our dataset. The wavelengths that most significantly contributed to group separation, identified as the top ten discriminatory bands based on PCA loadings and between-group effect size, are 650.8 nm, 661.4 nm, 665.9 nm, 589.5 nm, 659.9 nm, 667.4 nm, 730 nm, 662.9 nm, 664.4 nm, and 681 nm. Furthermore, three narrow spectral ranges—450–460 nm, 580–590 nm, and 700–800 nm—repeatedly exhibited disease-related variation and appear to be among the most informative features identified across the analyses. The identified ranges include significant pigment- and microbe-related inflection zones in both mean and derivative spectra, corresponding to the noted microbial and physiological changes in affected colonies. We recommend these specific wavelengths as potential diagnostic bands for future sensor design and field surveys, while emphasizing that the final operational selection should be guided by instrument spectral resolution and site-specific calibration.

Discrepancies in bacterial populations associated with different coral health statuses were identified through the analysis of mean and second derivative spectral data. Spectral indicators in the visible and near-infrared spectrum, particularly in the 550–800 nm range, are essential for distinguishing pathogenic bacteria from their phototrophic or symbiotic counterparts. Hierarchical clustering and K-means analyses offered unsupervised validation of these groupings, consistently generating functionally coherent clusters that mirrored microbial ecological roles and health associations. Although some overlap was present, especially among transitional bacterial types, the overall structure demonstrated distinct spectral separation.

The proposed non-invasive, scalable reflectance-based classification method offers a new potential diagnostic tool for the early identification of coral disease. In biodiversity hotspots like the Red Sea, where microbial dynamics are crucial for disease outbreaks and coral resilience, this strategy could be applied to reef-monitoring programs and remote sensing workflows.

## Supplementary Information

Below is the link to the electronic supplementary material.

Supplementary Material 1

Supplementary Material 2

Supplementary Material 3

Supplementary Material 4

Supplementary Material 5

Supplementary Material 6

Supplementary Material 7

## Data Availability

The datasets used and/or analyzed during the current study are available from the corresponding author on reasonable request.

## Abbreviations

PCA: Principal component analysis
HCA: Hierarchical cluster analysis
CCA: Canonical correspondence analysis
QS: Quorum sensing
FOV: Standard field of view
PTFE: Polytetrafluoroethylene
R: Reflectance
WPD: White plague disease
WBD: White band disease
NIR: Near-infraredred
